# Maternal natural killer cells at the intersection between reproduction and mucosal immunity

**DOI:** 10.1038/s41385-020-00374-3

**Published:** 2021-04-26

**Authors:** Evgeniya V. Shmeleva, Francesco Colucci

**Affiliations:** 1 Department of Obstetrics & Gynaecology, University of Cambridge, National Institute for Health Research Cambridge Biomedical Research Centre, Cambridge, CB2 0SW UK; 2 Centre for Trophoblast Research, University of Cambridge, Cambridge, UK

## Abstract

Many maternal immune cells populate the decidua, which is the mucosal lining of the uterus transformed during pregnancy. Here, abundant natural killer (NK) cells and macrophages help the uterine vasculature adapt to fetal demands for gas and nutrients, thereby supporting fetal growth. Fetal trophoblast cells budding off the forming placenta and invading deep into maternal tissues come into contact with these and other immune cells. Besides their homeostatic functions, decidual NK cells can respond to pathogens during infection, but in doing so, they may become conflicted between destroying the invader and sustaining fetoplacental growth. We review how maternal NK cells balance their double duty both in the local microenvironment of the uterus and systemically, during toxoplasmosis, influenza, cytomegalovirus, malaria and other infections that threat pregnancy. We also discuss recent developments in the understanding of NK-cell responses to SARS-Cov-2 infection and the possible dangers of COVID-19 during pregnancy.

## Introduction

**Fig. 1:**
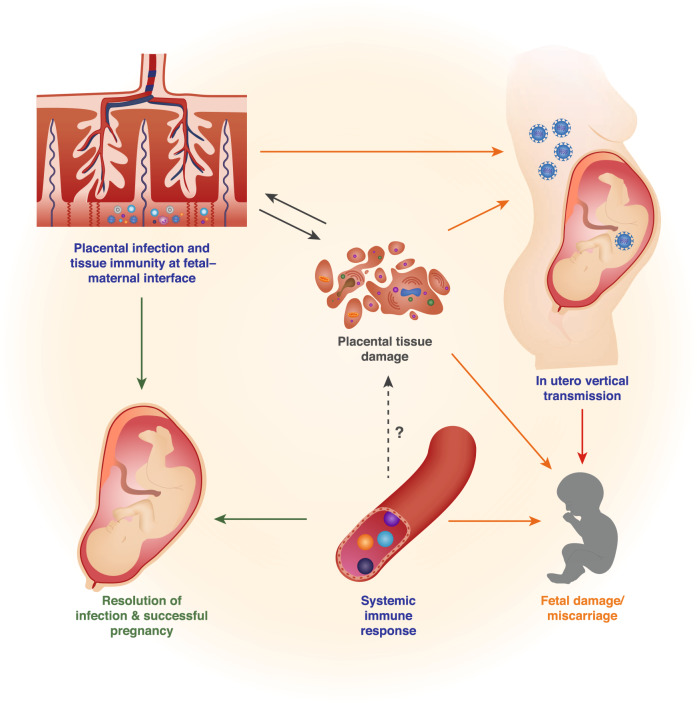
Influence on pregnancy outcomes by immune response to pathogens. Several aspects of the pathogenesis of a given infection can influence pregnancy outcomes: (i) tissue immunity to pathogen invasion at the fetal-maternal interface, (ii) in utero vertical transmission, and (iii) systemic immune response to infection. Both systemic and local tissue immune responses can be triggered by microbial invasion and may lead to pathogen elimination, and thus protect both mother and fetus. However, the activation of the immune system and the infection per se can lead to placental tissue damage, which might facilitate in utero vertical transmission and/or placental failure leading in turn to adverse pregnancy outcomes.

**Fig. 2:**
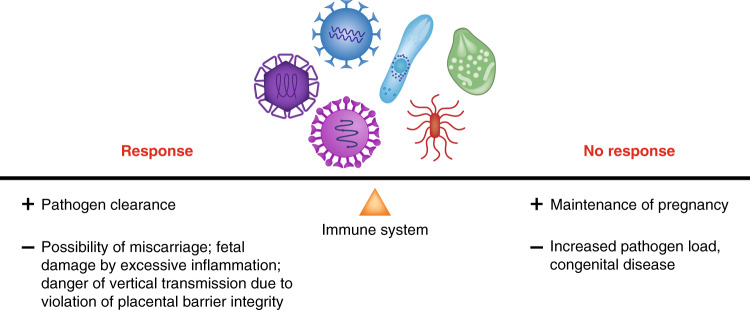
Double-edged sword: immune response during pregnancy. During infection, the maternal immune system has to balance between sustaining the growth of the fetus and protecting both mother and fetus from pathogens.

The vulnerability of pregnant women to infectious disease has been explained historically by assuming that the immune system weakens during pregnancy so that it can tolerate the fetus. Indeed, the maternal immune system does not reject the fetus or its placenta despite being aware of fetal antigens and responding to them, as illustrated by antibodies to paternal Rhesus or HLA antigens,^1^ or by male antigen-specific T cells found in the mothers of sons.^2^ Recent research is painting a more detailed picture of the immunological changes that occur during pregnancy, challenging the view of immunosuppression during pregnancy and revealing that, surprisingly, some aspects of immune responses are even stronger in pregnancy.^3,4^ The acronym TORCH refers to a set of pathogens (Toxoplasma, Other agents, Rubella, Cytomegalovirus and Herpes Simplex) that can cause abortion, intrauterine fetal growth restriction (FGR) and congenital infection. HIV, Malaria, and Influenza also cause severe adverse pregnancy outcomes. Complications of pregnancy due to infectious disease can be due to systemic or local factors, or to vertical transmission (Fig. 1). Immune responses to the pathogen may actually cause pregnancy complications. Complications may also occur without the pathogen crossing the placenta, as demonstrated by placental malaria when infected erythrocytes become sequestered in the placenta, triggering a syndrome similar to pre-eclampsia, the hypertensive disorder of pregnancy. With a focus on NK cells, we review the literature on pregnancy complications caused by major infections, including TORCH, malaria, influenza, ZIKA, and, possibly, SARS-Cov-2. NK and other immune cells may affect reproduction during implantation, gestation or parturition. We limit our discussion mainly to gestation, as NK cells are not key players during other stages of pregnancy (Fig. 2).

## Double-edged sword: NK-cell activation during infections in pregnancy

### NK cells

Peripheral blood NK (pbNK) cells play a substantial role against viral infections. Viruses have evolved numerous mechanisms to escape NK immunity by targeting their ligand-receptor pairs, signaling pathways, chemokines, and cytokines affecting NK-cell activation and recruitment.^5^ In return, NK cells respond by: (1) the recognition of “missing self” that viruses cause by downregulating MHC class I to escape T-cell responses; (2) activating receptors that sense host- and pathogen-derived ligands such as KIRs in humans, Ly49s in mice and, in both species, NKG2C, NCRs, and NKG2D; (3) antibody-dependent recognition via FcγRIII (CD16); (4) responding to IL-12, IL-15, and IL-18 and other cytokines produced by other cells.^6^ We review mechanisms of both direct and cytokine-induced NK-cell activation.

### Direct NK-cell activation

*KIR* are a set of variable genes (each individual inherits 4–20 genes from each parent) that code for inhibitory or activating receptors expressed in a variegated manner on subsets of NK cells and some T cells. Inhibitory and some activating KIR bind to HLA class I molecules, while other activating KIR bind to pathogen components within HLA class I molecules.^7,8^ Generally, inhibitory KIR suppress, while activating KIR enhance NK-cell functions such as cytotoxicity and cytokine production. However, inhibitory receptors can also promote NK-cell activation though the process of NK-cell education, which, broadly speaking, is the acquisition of functional competence through the interaction of inhibitory receptors with self MHC molecules.^9^ Each *KIR* gene can have dozens of allelic variants. Certain combinations of *KIR* and *HLA* genes associate with the outcome of HCV, HIV, and HCMV infections.^10–15^ Trophoblast expresses a unique combination of HLA class I molecules, including non-classical HLA-E, and -G, but does not express class II molecules. While HLA-A and -B are not expressed, the only polymorphic class I on trophoblast is HLA-C. Many studies link increased ability to control infection with either weak inhibitory or activating KIR variants,^16^ suggesting that enhanced activation of pbNK cells is beneficial for the host. Similarly, decidual NK (dNK) cells may require sufficient activation.^17^ Pre-eclampsia is positively associated with combinations of *KIR* and *HLA* that favor inhibition and is negatively associated with combinations that favor dNK-cell activation.^18^ Women pregnant with a *HLA-C2* fetus have a lower chance of developing pre-eclampsia,^19^ presumably because KIR2DS1^+^ dNK cells are activated upon ligating with fetal HLA-C2, and facilitate placental development.^20^ *KIR* and *HLA* variants may also influence responses to infection in pregnancy, and dNK cells can be induced to destroy pathogen-infected cells.^21^ HCMV infection of human decidual stromal cells (DSC) results in significant temporal downregulation of HLA-C expression; however, the expression of HLA-C is restored during the late stage of infection, which enables the activation of NK cells through KIR2DS1.^22^ Maternal KIR2DS1^+^ dNK cells, as well as pbNK cells, exhibit higher cytotoxicity to HCMV-infected DSC, especially to HLA-C2^+^ cells. Interestingly, dNK, but not pbNK cells, failed to secrete pro-inflammatory cytokines and degranulate in response to HCMV-infected HLA-G + EVT.^22^ KIR2DS1-mediated NK-cell activation might require modification of HLA-C by HCMV,^23^ so KIR2DS1-mediated dNK-cell degranulation may occur only against HCMV-infected cells. HLA-G engages KIR2DL4 on dNK cells and LILRB1 on myeloid cells and some dNK cells, and may have the tolerogenic function.^24^ HLA-G may also help infected and cancer cells to escape immunosurveillance.^25,26^ Soluble HLA-G (sHLA-G) levels in the amniotic fluid of *Toxoplasma gondii-*infected pregnant women were greater in the group with congenital infection than in the group without vertical transmission.^27^ This illustrates the conflict of the maternal immune system between sustaining the growth of the fetus and protecting it from pathogens. Higher sHLA-G levels might modulate the immune response and prevent fetal loss but can also lead to congenital infection. HLA-G may supress NK cells,^28,29^ although it can induce cytokine and chemokine production through interaction with KIR2DL4^30–32^ or LILRB1.^33^ HLA-E is expressed by trophoblast, maternal leukocytes, and stromal cells, where it interacts with activating CD94/NKG2C, expressed by a minority of pbNK and dNK cells and, preferentially, with inhibitory CD94/NKG2A, expressed by roughly 50% of pbNK cells and >90% dNK cells. The expression of HLA-E depends on peptides derived from other MHC class I molecules. While it may suppress dNK functions,^34^ NKG2A may also promote dNK-cell functions by enhancing NK-cell education.^35^

Natural Cytotoxicity Receptors (NCRs) on NK cells interact with host—as well as pathogen-derived ligands such as B7-H6, viral hemagglutinins, hemagglutinin neuraminidases, and other pathogen components.^36,37^ Among NCRs, NKp46 activates NK cells, while splicing variants of NKp44 and NKp30 mediate either activation or inhibition. While dNK cells predominantly express inhibitory isoforms, pbNK express activating isoforms.^38^ The switch from activating to inhibitory NKp44 and NKp30 is associated with decreased cytotoxicity and is induced by exposing pbNK cells to cytokines present in the decidual microenvironment, such as IL-15, IL-18, and TGF-β.^38^ Degranulation, target cell lysis or the production of pro-inflammatory cytokines and chemokines by dNK cells can be induced via NKp46 or NKp30 stimulation and abrogated by co-engagement of NKG2A.^39^ The only NCR in mice is NKp46; mice lacking NKp46 do not survive influenza A viral (IAV) infection.^40^ Interestingly, NKp46-deficient mice show defective uterine vasculature adaptations to pregnancy.^41^

NKG2D is an activating receptor expressed on the majority of pbNK cells and dNK cells in both humans and mice. It binds ligands normally absent on healthy cells and induced on stressed cells, therefore alerting immune cells. While these ligands are not found on trophoblast,^42^ they can be induced on uterine stromal cells and/or produced by trophoblast in soluble forms to interact with both dNK cells^43^ and pbNK cells.^44^ In a mouse model, trophoblast-expressed NKG2D ligand engaged NKG2D on NK cells, leading to the production of IFN-γ, which, in mice, is essential for uterine vascular remodeling.^45^ NKG2D is also involved in responses to infectious pathogens, as discussed below.

NK cells can shape memory immune responses.^46^ Human cytomegalovirus (HCMV)-seropositive individuals have an expanded CD94/NKG2C^+^ pbNK cell population^47^ and, in mice, Ly49H^+^ spleen NK cells amplify in response to mouse cytomegalovirus (MCMV) and protect against re-infection.^48^ Interestingly, during pregnancy, NKG2C^+^ dNK cells with enhanced IFN-ɣ and VEGF*α* production expand and may play functional roles in subsequent pregnancies.^33^

### Cytokine-induced NK-cell activation

Cytokines are essential for normal pregnancy but can also induce miscarriage, preterm labor, stillbirth, infertility, congenital fetal diseases and even behavioral changes in offspring.^49,50^ In mouse models, the administration of IFN-ɣ or poly(I:C) (a synthetic dsRNA which mimics viral infection and stimulates innate immune responses) to dams leads to fetal resorption,^51^ with the latter also causing increased serum TNF-α and upregulation of NKG2D expression on dNK cells and its ligand Rae-1 on F4/80^+^ decidual macrophages.^52^ The fetal resorption caused by poly(I:C) is prevented by depletion of dNK cells or by either neutralizing TNF-α or blocking NKG2D.^52^ Lipopolysaccharide (LPS), a component of the outer membrane of Gram-negative bacteria, also leads to fetal resorption and preterm labor. Interestingly, while *Ifng*^*−/*−^ dams are more resistant to LPS-induced resorption,^53^ *Il10*^*−/−*^ dams undergo preterm labor with low LPS doses that do not induce preterm labor in WT dams. LPS-induced preterm labor in *Il10*^−*/−*^ dams is associated with increased cytotoxicity of dNK cells and is prevented by the depletion of NK cells, neutralization of TNF-α, or recombinant mIL-10, which also restores low dNK cytotoxicity.^54^ Thus, dNK cells and macrophages, TNF-α, IFN-ɣ, and the NKG2D pathway are potential components of cytokine-induced pathogenesis of pregnancy complications that may be regulated by IL-10. These studies also illustrate how dNK cells, key players in the physiology of pregnancy, can mediate fetal resorption if the decidual milieu is perturbed. Similarly, in a mouse model of fetal/neonatal alloimmune thrombocytopenia, antibody-dependent cellular cytotoxicity (ADCC) toward anti-β3 integrin resulted in decidual infiltration of NK cells and trophoblast apoptosis, leading to miscarriage and intrauterine FGR, which could be prevented by NK-cell depletion or by blocking NKp46 or FcɣRIIIa receptors.^55^ While these are clear examples of dNK cells causing pregnancy complications in pathological situations, there is no clear evidence that pbNK and dNK are implicated in recurrent miscarriage or implantation failure in humans.^56^

Table 1 summarizes the known roles of dNK and pbNK cells in human pregnancy complications and mouse models. In the following sections we review these roles in individual infectious diseases.

**Table 1:** Role of decidual or peripheral blood NK cells in human pregnancy complications and mouse models.

Pathogen	Pregnancy complications	Resemblance of mouse model to human pathology	Role of NK cells in infection during pregnancy		
			Role	References	Cell
*Toxoplasma gondii*	Miscarriage, stillbirth, vertical transmission, congenital toxoplasmosis (chorioretinitis, hydrocephalus, intracranial calcifications, blindness, deafness, others)	Acute toxoplasmosis in immunocompetent humans is generally asymptomatic while experimental infection in mice may be severe and even fatal. However, pregnant animals can display miscarriage (resorption), vertical transmission and low birth weight upon acute *T. gondii* infection. There are many strains of *T. gondii* with different virulence as well as several route and parasite stage options for infection used for animal experiments, which influence the severity of infection and observed phenotypes	Apoptosis of trophoblasts	^67^	dNK
			Cytotoxicity toward *T.* gondii-infected cells	^70,72,86^	dNK
			IFNɣ production in response to *T. gondii* infection	^70,86^	dNK
			INFɣ-mediated decrease of parasite load and materno-fetal transmission (comparison between WT, *Ifnɣ*^*−/−*^ and anti-IFNɣ antibody-treated mice)	^76,78^	NK*
			INFɣ-mediated miscarriage (comparison between WT and *Ifnɣ*^*−/*^^−^ mice)	^78^	NK*
Influenza A Virus (IAV)	Higher risk of severe influenza infection in pregnant women in comparison with non-pregnant individuals	The severity of IAV infection in mice depends on the dose and the virus strain. Mortality rates in pregnant mice are significantly higher than in non-pregnant animals, which resembles human data	Increased NK cytotoxicity as well as IFNɣ and other cytokine/chemokine production in response to IAV in pregnant individuals then in non-pregnant counterparts	^103,104^	pbNK
*Plasmodium falciparum*	Higher susceptibility and more severe malaria in pregnant individuals, low birth weight, miscarriage, preterm labor, stillbirth, vertical transmission (rare) and congenital malaria (rare)	Mouse models are far from ideal for studying malaria infection, as rodent-specific parasite species have to be used. However, the accumulation of plasmodium in placenta, placental inflammation and tissue damage, dysregulation of cytokine levels, lower survival rates of dams, resorption, intrauterine growth retardation and stillbirth can be observed in mouse models. Also, humanized mouse models can be applied to study *P. falciparum*; however, it is still unknow how well these models represent *P. falciparum* infection*-*associated pathophysiology	INFɣ-mediated decrease of parasite load (comparison between WT and *Ifnɣ*^*−/*^^−^ mice; also, results (correlation only) from observational study on humans)	^130,136^	NK*, pbNK*
			INFɣ-mediated miscarriage (comparison between WT and *Ifnɣ*^*−/*^^−^ mice)	^136^	NK*
*Listeria monocytogenes*	Higher susceptibility to listeriosis in pregnant individuals, pregnancy loss, preterm birth, stillbirth, vertical transmission and congenital diseases	Listeriosis is a natural infection for rodents; however, susceptibility to *L. monocytogenes* varies a lot between different strains of mice. Spontaneous abortion (resorption), placentitis, placental necrosis, endometritis, stillbirth and vertical transmission are described for listeriosis in mice, but mechanisms of the placental barrier crossing differ between WT mice and humans	Killing of L. monocytogenes by injecting anti-microbial pertide granulysin through nanotubes to infected cells	^148^	dNK, pbNK
Hepatitis C Virus (HCV)	Vertical transmission	Mice are not susceptible to HCV. There are xenograft and humanized mouse models, but they are designed to study liver disease and not pregnancy outcomes; therefore, they are not suitable for mother-to-fetus transmission	–		
Human Immunodeficiency Virus (HIV)	Vertical transmission, infants born from HIV-positive women are at higher risk of intrauterine FGR and low birth weight	Mice are not susceptible to HIV. There are several humanized mouse models; however, the relevance of these models for pregnancy complication research is not described	Inhibition of HIV infection	^213^	dNK
Zika Virus (ZIKV)	Vertical transmission, fetal/infant central nervous system abnormalities such as microcephaly and other neurological disorders	WT mice are resistant to ZIKV; however, mice lacking IFN type I production or the ability to respond to it are susceptible. Placental infection, vertical transmission and fetal pathological changes after ZIKV infection were shown in *Ifnar1*^*−/−*^ mice	–		
Rubella Virus (RV)	Among major pregnancy complications are miscarriage, stillbirth, congenital disease, and a wide spectrum of fetal abnormalities such as cataract, hearing loss, cardiovascular and central neural system defects	There is no suitable mouse model	–		
Cytomegalovirus (CMV)	Vertically transmitted in utero, leading to a variety of disorders including hearing and vision loss, intracranial calcifications, and mental retardation	Mice are not susceptible to HCMV; MCMV infection is used as a model to study cytomegalovirus infection in pregnant mice, where the reductions of placental and fetal brain weights were described. However, MCMV in utero vertical transmission in mice does not occur. A human placental villi xenograft SCID mouse model can be used to study some aspects of HCMV placental infection, but not pregnancy outcomes	Cytotoxicity toward HCMV-infected cells	^260^	dNK
Herpes Simplex Virus (HSV)	No enough evidence	Susceptibility to HSV depends on the virus strain and the mouse background. Medroxyprogesterone-treated WT mice can be infected intravaginally with HSV-2	–		
Other Herpesviruses	Spontaneous abortion (HHV-6/7), pre-eclampsia (iciHHV-6)	There is no suitable mouse models to study HHV-6/7	Cytotoxicity toward HHV-6A infected cells (endometrial NK)	^276,277^	dNK
SARS-Cov-2	Vertical transmission and other pregnancy complications cannot be excluded and requires further investigation	Several mouse models are proposed to study SARS-CoV-2 infection (hACE2 transgenic mice, adeno associated-hACE2 virus mouse model and the infection of WT mice with mouse-adapted SARS-CoV-2). There is currently no data available on the adverse pregnancy outcomes in SARS-CoV-2-infected mice	–		

“–” No data on role of NK cells in pregnancy for this pathogen.

“*” Indirect finding/conclusion.

## Major infections during pregnancy: TORCH (Toxoplasmosis, Other agents, Rubella, Cytomegalovirus, Herpes Simplex)

### Toxoplasmosis

Vertical transmission occurs in 30–40% of toxoplasmosis during pregnancy, with a 10–80% risk of congenital diseases in early and late gestation, respectively.^57,58^ Human syncytiotrophoblast is not permissive to infection by *T. gondii*, while cytotrophoblast, placental fibroblasts, chorion, and amnion are. In a small cohort of pregnant women, vertical transmission was associated with elevated INF-ɣ and decreased TGF-β in supernatants from peripheral blood mononuclear cells (PBMC) stimulated with soluble *T. gondii* antigen.^59^

In response to *T. gondii* infection, NK cells help to recruit and differentiate monocytes, macrophages, T lymphocytes, and dendritic cells (DCs) via NKG2D and IFN-ɣ,^52,60–63^ a key cytokine during toxoplasmosis.^64^ Indeed, animal experiments demonstrated that *T. gondii* infection leads to high expression of genes related to type I and especially type II interferon pathways in blood and lungs.^65,66^ Activation of dNK cells in response to *T. gondii* may contribute to tissue pathology during pregnancy: co-incubation of *T. gondii*-infected human dNK cells with trophoblast resulted in increased caspase 3 and 8 mRNA and trophoblasts apoptosis, which were reduced by IFN-ɣ neutralizing antibodies^67^ or IL-10, which also upregulated the apoptosis inhibitor c-FLIP in infected primary human trophoblasts.^68^

Animal experiments and in vitro studies with human dNK cells showed that IL-12 and TNF-α are essential for NK-cell stimulation and defense against *T. gondii*.^69,70^ Human and murine DC and DC-derived IL-12 facilitated IFN-ɣ production by NK cells^61,69,71^ and decidual DC-derived IL-12 enhanced NKG2D-mediated dNK cytotoxicity toward *T. gondii*-infected cells.^70^ Cytotoxicity of human dNK was increased when incubated with *T. gondii*-infected trophoblast cells.^72^ *T. gondi* infection enhanced the expression of *NKG2D*, *KIR2DL4,* and *LILRB1* mRNA in dNK cells and *HLAG* in trophoblast cells.^72^ In vivo experiments on pregnant mice also demonstrated increased expression of NKG2D as well as NKG2A on dNK after *T. gondi* infection.^72^

Most information about immunopathogenesis of acute toxoplasmosis during pregnancy comes from mouse models. However, acute toxoplasmosis in immunocompetent humans is generally asymptomatic, while experimental infection in mice may be severe or even fatal.^73–75^ Generally, *T. gondi*-infected dams abort^72^ and most experiments suggest a critical role for NK-cell derived IFN-ɣ.^61,76–78^ Indeed, *T. gondii* infection of *Ifnɣ*^−*/−*^ mice or NK-cell-depleted WT mice resulted in increased parasite load.^65,79^ Type I IFNs also participated in controlling *T. gondii* infection and, in its absence, blood and liver parasite burden increased,^66^ while recombinant IFN-β protected animals from lethal infection.^80^ Also, enhanced NK-cell-mediated and perforin-independent^81^ cytotoxicity was demonstrated in response to *T. gondii* compounds.^82,83^ Despite higher parasite load in *Ifnɣ*^−*/*−^ dams, all mice delivered live pups, whereas only a fraction of infected WT dams gave birth to live pups, varying from 5–10% to 40–60% depending on whether infection occurred during the first or second half of pregnancy, respectively.^78^ Nonetheless, in comparison with WT dams, *Ifnɣ*^−*/*−^ mothers died and when their pups were fed by infected WT foster mothers, *Ifnɣ*^*−/*−^ pups had significantly lower body weight and died by the age of 6 weeks.^78^ Interestingly, after Toxoplasma infection, dNK cells declined in *Ifnɣr*^−*/*−^ but not in WT dams.^84^ These experiments demonstrate that IFN-γ signaling in mice is key to controlling parasite loads in blood and uterus, but not conducive to progression of pregnancy and live birth.^84^

High fetal loss and the reduction of fetoplacental weight were also found in dams infected with a less lethal strain of *T. gondii* (mutant Wh3Δrop16). The escalation of infection with this mutant strain was associated with the production of pro-inflammatory cytokines such as IL-12, IL-17, IFN-ɣ and reduced secretion of IL-10, IL-4, and TGF-β in the placenta.^85^ Treatment of *T. gondii*-infected dams with TGF-β decreased resorption rates, improved fetal weights and diminished inflammation.^86,87^ This improved pregnancy outcome was linked to a TGF-β-mediated reduction of dNK-cell cytotoxicity and IFN-ɣ production.^86^

Blood and liver NK cells are also the main source of IL-10 in acute toxoplasmosis: IL-10 production is induced by systemic IL-12 during infection dissemination. Animal experiments demonstrated that NK cells participate in a negative feedback loop by IL-10 secretion, which leads to reduced IL-12 production.^88^ NK cells can restrict pathogen clearance via IL-10 secretion: *T. gondii* burden was significantly decreased in mice with impaired NK-cell IL-10 production.^89^

Interestingly, *Il15*^*−/−*^ mice survive *T. gondii* infection that is lethal to WT mice. The successful survival of *Il15*^−*/−*^ mice was not due to better ability to clear the pathogen, but related to less severe intestinal inflammation, which was in turn associated with impairment of NKp46^+^NK1.1^+^CD127^−^ group 1 Innate Lymphoid Cell (ILC1)-mediated recruitment of inflammatory monocytes via secretion of CCL3 and the inability of inflammatory monocytes to induce damage of the small intestine. In this model, ɣδ and CD8^+^ T cells did not influence the course of infection.^79^ In another study, ILC1s were potent IFN-ɣ and TNF producers and participated in the control of *T. gondii* infection.^90^ Mice lacking the ILC1-master transcription factor T-bet or alymphoid *Rag2*^*−/−*^ *Il2rɣ*^*−/−*^ mice had higher *T. gondii* loads than WT animals and recruited less inflammatory monocytes into the small intestine.^90^ Adoptive transfer of ILC1s into *Rag2*^*−/−*^ *Il2rɣ*^*−/−*^ mice decreased parasite burden and significantly enhanced monocyte infiltration. RNAseq analysis of blood and lungs obtained from mice at 7 days post infection with *T. gondii* showed signatures of increased NK cells.^66^ However, other studies demonstrated a decrease of NK cells in spleen of mice after *T. gondii* infection,^65,91^ while splenic ILC1s increased due to a conversion of NK cells into ILC1-like cells.^65^ After *T. gondii* infection of mice, NK and ILC1-like cells had increased expressions of KLRG1, DNAM-1, Neuropilin-1, CXCR8, and CCR8.^65^

Taken together, in vitro experiments with human cells and in vivo mouse models suggest that type I INF, IFN-γ and TNF-α are key to controlling *T. gondii* infection, however, activation of DC, IL-12 and IL-15 may lead dNK cells to damage trophoblast, and this may be counteracted by IL-10 and TGF-β.

### Other infectious agents

#### Influenza

Pregnancy is a risk factor for severe influenza.^92–95^ Influenza virus does not infect or cross the placenta, however, the elevation of some systemic cytokines, especially type I and II IFNs, TNF-α, IL-1β, IL-6, and IL-15 may cause collateral damage.^96–99^

There is evidence that either a lack of NK-cell function or NK-cell hyperactivity contribute to influenza immunopathology during pregnancy. Three patients with severe H1N1/09 influenza had low pbNK cell counts, with one lethal case of a pregnant women with IAV viremia, scarce infiltration of lung tissues with immune cells and a total absence of NK cells in lung samples.^100^ IAV could also directly infect human pbNK cells, induce their apoptosis, and downregulate NKp46- and NKp30-mediated cytotoxicity.^101,102^ Conversely, upon co-incubation of pbNK with H1N1-infected autologous monocytes, pbNK cells isolated from pregnant individuals produced higher levels of IFN-ɣ and degranulated more than pbNK cells isolated from control women. Moreover, the expression of NKp46 and CD38 were also increased during pregnancy.^103^ In addition, pbNK, CD4, and CD8 T cells obtained from pregnant individuals at day 7 post vaccination with the seasonal vaccine had more robust responses to IAV infection than cells from non-pregnant vaccinated women.^104^

Mouse models support clinical observations and demonstrate more severe influenza infection in pregnant than in non-pregnant animals.^105–107^ In comparison with non-pregnant infected mice, dams had similar counts of lung-infiltrating NK cells^108^ but higher IL-1β, IL-6, CCL3, and CXCL2, while IFN-ɣ was lower in lungs.^105^ Also, BALs of IAV-infected dams had higher levels of pro-inflammatory cytokines/chemokines and contained greater counts of macrophages and neutrophils.^106^ LPS causes preterm birth in a dose-dependent manner, and IAV primes dams to preterm birth with lower LPS doses.^109^ Type I IFN signaling was key for the priming effect of IAV, which was also observed for LCMV and listeriosis. WT dams injected with IL-6 neutralizing antibodies or *Ifnar*^*−/−*^ dams did not exhibit preterm birth.^109^ The “double-hit” hypothesis suggests that viral infections predispose the organism to subsequent bacterial infection, leading to more severe disease.^110–112^

Similar to humans, mouse NK cells may also be protective or contribute to the immunopathology of severe influenza. While NKp46-deficient mice were more susceptible to influenza,^40^ *Il15*^*−/*−^ mice, as well as NK-cell-depleted WT mice, were resistant to IAV infection in comparison with WT mice. The resistance was not related to viral clearance, as IAV titers in lungs were similar in all groups.^113–115^ Interestingly, leukocyte infiltration and productions of IL-6 and IL-12 were decreased, but IL-10 increased in bronchoalveolar lavage of *Il15*^−*/*−^ mice, and in NK-cell-depleted mice.^113^ In addition, TNF-α and IL-6 serum levels, as well as lung NK- and CD8 T-cell infiltration were decreased in *Il15*^*−/*−^ mice.^114^

#### Malaria

Women without pre-existing immunity to *Plasmodium falciparum* are at a greater risk of maternal and perinatal mortality. Common complications of pregnancy during malaria are low birth weight, miscarriage, preterm labor and stillbirth.^116–118^ Pathogenesis of malaria during pregnancy is a complicated process which includes erythrocyte lysis, hormonal changes (e.g., cortisol release), red blood cell (RBC) accumulation, intravascular thrombosis, and immune responses.^119,120^ NK cells contribute significantly to immune responses to malaria.^121,122^ Human pbNK cells form conjugates with infected RBC and lyse them via ADCC.^123,124^ Co-incubation of human PBMCs with live *P. falciparum* erythrocytic schizonts induced IFN-ɣ production and more than 50% of IFN-ɣ^+^ cells were pbNK cells.^125,126^ Interestingly, the association of placental malaria with certain *KIR* genes is modified by co-infection with HIV and CD4 T-cell counts, suggesting co-operation between NK and T cells in response to *P. falciparum*.^127^

INF-ɣ plays dual roles in malaria pathology, being crucial for parasite clearance, but also responsible for disease severity and cerebral malaria.^128^ Expression of *IL-1β*, *IL8*, and *TNFA* mRNA were significantly increased while *IL-6* and *TNFB* were lower in placentas of women with malaria. *TNFA* correlated with placental hemozoin, a marker of malaria infection; and *TNFA* and *IL8* correlated with intrauterine FGR.^129^ Women with placental malaria had higher peripheral parasitaemia and displayed lower IFN-ɣ production by NK as well as by CD4 and CD8 T cells, in response to in vitro stimulation with PMA/ionomycin/IL-2.^130^

Animal models have been widely used to study malaria infection during pregnancy. Infected pregnant rhesus macaques had detectable serum TNF-α and lost their fetuses, however, animals with no detectable serum TNF-α delivered live babies.^131^ Similar to humans and monkeys, pregnant mice experienced more severe malaria and pregnancy complications than non-pregnant infected counterparts.^132,133^ Upon infection with *Plasmodium chabaudi*, *Ifnɣ*^−/−^ or *Ifnɣ*r^−/−^ animals had greater mortality rates, along with significantly higher parasite loads, lower serum concentrations of IL-12 and nitric oxide, diminished numbers of splenic macrophages and NK cells, as well as higher serum concentrations of IL-10.^134,135^ IFN-ɣ and TNF are responsible for fetal loss in plasmodium-infected mice. Despite *Ifnɣ*^−/−^ dams experiencing higher parasitaemia and more pronounced anemia, fetal loss was delayed in comparison with WT dams.^136^ Plasma TNF was increased in infected dams and anti-TNF treatment decreased fetal loss, despite having no effect on parasite load.^136^

#### Listeriosis

Pregnant women are more susceptible to infection with *Listeria monocytogenes*, which is more common in the third trimester and can lead to pregnancy loss, preterm birth, stillbirth, vertical transmission, and congenital diseases.^116^ Transmitted via oral route, *L. monocytogenes* crosses the intestinal barrier, disseminates to placenta via blood and then passes the placental barrier which results in fetal infection in at least 25% of cases.^137^ *L. monocytogenes* infects extravillous trophoblasts (EVT) and syncytiotrophoblast where two bacterial internalins, InlA (a ligand for E-cadherin) and InlB (a ligand for c-Met), independently play critical roles in fetoplacental listeriosis.^137,138^ While neutrophils, monocytes, and macrophages play important roles in the innate immunity to listeria,^139–141^ NK cells are also involved. Most of our knowledge comes from mouse models. Similar to other acute infection diseases after *L. monocytogenes* infection, *Ifnɣ*^−*/*−^ or WT mice treated with anti-IFN-ɣ blocking antibodies have a greater bacterial burden than control groups.^142,143^ NK cells are the main source of IFN-ɣ during listeriosis.^144,145^ Also, NKp46^+^RORɣt^-^ ILC1s and NKp46^+^RORɣt^+^ ILC3s secreted IFN-ɣ and IL-22, respectively, in the small intestine and mesenteric lymph nodes of orally infected mice.^143^ Interestingly, splenic and hepatic bacterial loads were lower in *Il15*^*−/*−^ or NK-depleted mice, which also survived at a greater rate than WT or sham-treated mice,^144,146,147^ and no difference was seen between *Il15*^−*/*−^ and WT-infected dams, suggesting that NK cells are not required for protection against *L. monocytogenes* in either pregnant or non-pregnant mice, and can even be detrimental for pathogen clearance.^146^ However, very recently, dNK cells were shown to kill *L. monocytogenes* from within EVT, by injecting anti-microbial Granulysin through nanotubes.^148^

NK cells may influence the response of myeloid cells to *L. monocytogenes* by secreting IL-10. Indeed, *L. monocytogenes* stimulated IL-10 production by mouse splenic, liver, and pbNK cells at 72 and 96 h post infection, but not at 24 h, which coincides with the peak of IFN-ɣ production by NK cells. Bacterial burden was lower in infected *Il10*^−*/*−^ and NK-depleted mice; moreover, both *Il10*^−*/*−^ and anti-NK1.1 treated animals had increased splenic neutrophil and monocyte numbers at 2–4 days post infection.^144^ Similarly, NK-cell IL-10 inhibited host resistance to *Leishmania donovani*.^149^ NK cells were the main IL-10 producer among splenocytes of mice infected with *L. donovani*; nonetheless, the early NK-cell response to leishmania was IFN-ɣ secretion, and NK cells switched to IL-10 production only at later time points of infection.^149^

#### Hepatitis C virus (HCV)

Acute HCV infection evolves into chronic infection in 75–80% of cases, with 10–20% of HCV-positive individuals resulting in cirrhosis and hepatocellular carcinoma after 20–30 years of virus persistence^150,151^ and the immune responses may both clear the virus and contribute to liver injury.^152,153^ Seventy-one million people globally are estimated to live with chronic hepatitis C.^154^ Although the rate of mother-to-fetus transmission is estimated to occur in 5–10% of cases,^155^ and the current etiotropic treatment is effective, HCV vertical transmission is a serious healthcare problem. Transmission is associated with the use of injection drugs, high viremia, PBMCs infection, HIV co-infection, the rupturing of membranes, and amniocentesis.^156–160^ HCV transmission may occur during delivery, while an estimated one third of infected babies may acquire HCV in utero.^161,162^ Although HCV may significantly impact pregnancy outcomes, the associations between the virus and low-birth weight, intrauterine FGR or preterm birth are still controversial^163–169^ because chronic hepatitis C can be accompanied by extrahepatic HCV-associated disorders, such as autoimmune diseases and low-grade inflammation.^170–174^

The role and activation status of pbNK and hepatic NK cells varies throughout the course of HCV infection and differs during early and later stages of liver fibrosis or cirrhosis,^153,175–177^ and both defensive and pathogenic roles are considered.^177^ As mentioned earlier, certain *KIR* and *HLA-C* variants have been associated with the resolution of hepatitis C.^16^

It is difficult to model chronic HCV disease and the course of pregnancy during HCV infection in small animal models.^178^ Therefore, it is unknown how HCV vertical transmission occurs and if immune cells at the fetal-maternal interface participate in the prevention of transmission. A small study with only five cases of vertical HCV transmission did not reveal signs of placenta pathology.^179^ Human villous cytotrophoblast express receptors for HCV uptake and therefore, can be infected by HCV,^180,181^ which results in upregulation of type I and III IFNs, CXCL11, and CXCL12, as well as potential dNK activation by HVC-infected trophoblast.^180^

#### Human immunodeficiency virus (HIV)

Around 38 million people worldwide are currently HIV positive.^182^ HIV has two major types: HIV-1 and HIV-2, where HIV-1 is the most common type and responsible for the pandemic.^183^ HIV infects CD4 T cells, macrophages and DC.^184^ Before the era of highly active antiretroviral therapy (HAART), HIV vertical transmission occurred in 15–40% of cases^185,186^ but HAART has reduced the perinatal transmission rate to <1%.^187–189^ The virus can be detected in maternal blood, vaginal fluid, and breast milk.^190,191^ Greater risk of mother-to-child transmission is associated with high maternal viral load in blood, low blood CD4 T-cell count, long duration of membrane rupture prior to delivery and breastfeeding.^192–195^ How HIV crosses the placenta is unclear. HIV can infect trophoblast cells in vitro, however, cell-associated HIV can also cross the placenta presumably by cell-to-cell contact and transcytosis.^196^ Although no specific histopathological abnormalities are demonstrated, there are indications of chorionitis^197,198^ and placental membrane inflammatory lesions in HIV-positive women, though these do not correlate with increased risk of vertical transmission.^199^ The above changes may be due to HIV-associated opportunistic infections. Elective cesarean section significantly reduces the risk of perinatal transmission to 2–10 % in women without antiviral therapy,^192,200–202^ suggesting that the majority of HIV perinatal transmission occurs intrapartum through contact with vaginal fluid or maternal blood.^196^ Infants born from HIV-positive women are at higher risk of intrauterine FGR and low-birth weight^203,204^; however, it is difficult to discern the effects directly caused by HIV from those caused by opportunistic infections and antiretroviral therapy.^205–208^

Most research on NK-cell responses to HIV infection is done using human pbNK cells. The role of NK cells in controlling HIV infection and disease has been reviewed, and is related to natural cytotoxicity, ADCC, cytokine/chemokine production, and their influence on adaptive immune responses.^209–211^ Low percentage of pbNK cells is associated with increased risk of HIV vertical transmission.^194,195^ There is evidence that dNK cells may participate in the protection of the fetus against HIV transmission. Indeed, uterine-derived NK (uNK) cells (but not pbNK cells) activated in vitro with IL-12 and IL-15, inhibited infection of cell lines, PBMCs, and primary human endometrial cells with strains of HIV that use CXCR4 for cell entry.^212^ The inhibitory activity was associated with uNK-produced CXCL12, a CXCR4 ligand, and supressed by CXCL12 neutralising antibodies.^212^ Moreover, dNK cells significantly delayed and reduced infection of decidual macrophages with HIV strains that use CCR5 for entry. and they did so via cell-cell contact and IFN-ɣ release.^213^

#### Zika virus (ZIKV)

The recent epidemic brought ZIKV to attention due to its involvement in the development of fetal/infant central nervous system abnormalities.^214^ Although ZIKV infections may result in fetal/infant abnormalities during any stage of pregnancy, the first trimester has the highest risk for adverse outcomes.^215,216^ ZIKV can infect a range of cells in decidua and placenta explants such as decidual fibroblasts and macrophages, trophoblasts, fetal macrophages known as Hofbauer cells, and umbilical cord mesenchymal stem cells.^217–221^ While trophoblast at early- and mid-gestation may be more susceptible to ZIKV,^220,222^ trophoblast from full-term placenta is relatively resistant to ZIKV infection, which may be due to its constant IFN-λ production.^223^ However, rather than in trophoblast, the virus predominantly replicates in Hofbauer cells,^224–226^ which respond by secreting IFN-α, IL-6, and CXCL10.^219^

Chemokines and cytokines increase modestly in the blood of patients with acute ZIKV infection.^227–229^ Amniotic fluid from pregnant women with microcephalic babies have significantly increased levels of IL-6, IL-15, IL-17, IFN-ɣ, and TNF-α, which have the potential to contribute to fetal neurological disorders.^50,230,231^ Ex vivo experiments on ZIKV-infected human decidual tissues have shown upregulation of type I and III IFN signaling pathways.^232^

Studies on the role of dNK cells during ZIKV infection have not yet been conducted. Co-incubation of human pbNK cells with ZIKV-infected cells did not activate NK-cell ligand expression on epithelium cells.^233^ In contrast, ZIKV led to IFNβ-mediated upregulation of MHC-I expression, which resulted in the inhibition of NK-cell killing; however, NK cells were still able to produce IFN-ɣ, but not TNF-α when co-incubated with ZIKV-infected cells.^233^ While both myeloid and lymphoid cell populations, including NK cells, were decreased in blood of patients with viremia in comparison with healthy donors and nonviremic patients,^228^ the percentages of blood NK and T cells expressing the Ki67 proliferative marker significantly increased in rhesus macaques after ZIKV infection of pregnant and non-pregnant animals.^234^ Vertical transmission and fetal pathology also occur in animal models of ZIKV infection.^216,235–237^ Increased IFN-β production and activation of IFN-stimulated genes were found in placentas of pregnant mice after intrauterine ZIKV infection.^238^ The importance of type I IFNs for protection against Zika virus was demonstrated in animal models, where *Ifnar1*^−*/*−^ mice are broadly used to model ZIKV infection.^216^ Placentas of mice infected with ZIKV during the first half of pregnancy had signs of trophoblast destruction and vascular damage; also, FISH analysis of placental sections revealed ZIKV RNA in different trophoblast cells.^236^

### Rubella virus (RV)

Rubella in pregnancy is associated with 85% risk of birth defects, especially if the maternal infection occurs in the first trimester.^239,240^ Vaccination has substantially reduced cases, but some are still reported and globally, around 100,000–200,000 babies are born with congenital rubella every year.^241^ RV infection is associated with major pregnancy complications including miscarriage, stillbirth, congenital disease, and a wide spectrum of fetal abnormalities such as cataract, hearing loss, cardiovascular and central neural system defects.^239,242^

RV can be found in any fetal/infant organ.^239^ RV-infected placentas display non-specific pathological changes similar to other viral infections, such as necrosis of trophoblast and endothelial cells, focal mononuclear and lymphocyte infiltration, fibrin deposition, interruptions of the syncytium and vasculitis.^243,244^ Studies on immune responses to RV ex vivo are lacking. RV infection of human fibroblast cell lines in vitro resulted in significant upregulation of interferon-stimulated genes.^245^ Pre-treatment of cell cultures with anti-type I IFN serum before RV infection led to increased viral titers and numbers of infected cells.^245^

### Cytomegalovirus (CMV)

NK cells play a key role in the control of herpesvirus infections. Patients with primary NK-cell deficiencies are susceptible to severe infectious diseases caused by varicella zoster virus, herpes simplex virus, cytomegalovirus, or Epstein-Barr virus.^246,247^ Among all herpesviruses, HCMV plays the most prominent role in pregnancy complications, with 30–50% of primary and 0.5–2% recurrent HCMV maternal infections vertically transmittable in utero, leading to a variety of disorders including hearing and vision loss, intracranial calcifications, and intellectual disability.^248–252^ Although infection during early pregnancy only rarely leads to congenital infection, it can lead to severe adverse pregnancy outcomes.^253^ The cellular tropism of HCMV is very broad, including epithelial and endothelial cells, smooth muscle cells, neurons, dermal fibroblasts, and myeloid cells.^74,254^ Examination of HCMV-positive placentas as well as ex vivo-infected decidual and villous explants revealed that HCMV is present in DCs, macrophages, endothelial cells, fibroblasts, cytotrophoblast, and rarely in syncytiotrophoblast, where it does not replicate.^255–258^

Immunohistochemical analysis of HCMV-positive placentas showed leukocyte infiltration and the presence of CD8^+^ T and CD56^+^ NK cells.^256,259,260^ Co-incubation of human dNK cells with HCMV-infected fibroblasts changed dNK receptor repertoire as well as their secretory profile, thus restoring cytotoxicity. Activating NKG2D and CD94/NKG2C/E receptors, but not NKp30 or NKp46, played crucial roles in killing HCMV-infected autologous decidual fibroblasts.^260^ HLA-E and MICA/B expressions on HCMV-infected DSC were not impaired, while HLA-C expression was downregulated. Because of this HLA-C downregulation, inhibitory KIRs on NK cells could no longer be engaged, and therefore killing of virus-infected DSC might occur via missing self-recognition.^261^ Accordingly, HLA-G expression on trophoblast is also impaired during HCMV infection,^255^ which might activate NK-cell cytotoxicity because the inhibitory LILRB1 receptor on NK cells is no longer engaged.^262^ However, the downregulation of HLA-G expression by EVT was not observed in a more recent study.^22^ It is important to note that protein and gene expression profiles of human dNK cells, as well as their ability to respond to infection, significantly differ through pregnancy. During incubation with HCMV-infected DSC, first trimester dNK cells degranulates similar to pbNK cells, while the degranulation capacity of term pregnancy dNK cells is significantly declined.^261^

Systemic immune responses to cytomegalovirus have also been described, where patients with acute symptomatic HCMV were found to have increased numbers of NK, T, and B cells in their blood.^263^ Although the expression of activating NKG2C, NKG2D, and CD38 as well as inhibitory NKG2A receptors were increased on pbNK, degranulation and IFN-ɣ production by pbNK was similar to control groups.^263^ Pregnant women with confirmed HCMV fetal infection had elevated serum CXCL10 and elevated amniotic fluid levels of TNF-α, IL-1β, IL-10, IL-12, IL-15, IL-17 and CCL2, CCL4, and CXCL10.^264^ HCMV infection increased *IFN-ɣ* and *CXCL10* mRNA in human decidual tissues and *CXCL10* in placental villi.^265^ Anti-TNF-α antibodies added to trophoblast cultures at the time of HCMV challenge significantly reduced trophoblast apoptosis.^266^

Acute murine cytomegalovirus (MCMV) infection of pregnant mice results in immune cell infiltration and focal necrosis of placental tissues as well as reductions of placental and fetal brain weights; however, vertical transmission of MCMV in mice does not occur.^267–269^

### Herpes simplex and other herpesviruses

NK cells have numerous tools to combat herpesviruses, which have evolved escape mechanisms to establish latent infection.^5,270,271^ Blood CD56^dim^ NK cells decrease in the second and third trimesters of pregnancy,^4^ and this may predispose pregnant women to herpesviruses. Despite two studies reporting pregnancy loss associated with HSV-infection in the first trimester of pregnancy,^272,273^ there is not enough evidence to conclude that HSV causes adverse pregnancy outcomes. The importance of NK cells for the control of HSV-2 infection was demonstrated in mouse models, using alymphoid *Rag2*^−*/*−^*Il2rg*^−*/*−^ and NK-deficient *Il15*^−*/*−^ animals, which did not survive intravaginal HSV-2 infection.^274^

The evidence for the involvement of HHV-6 and HHV-7 in pregnancy complications is clearer. HHV-6A DNA was expressed in endometrial samples of 43% of patients with idiopathic infertility, while no virus was detected in control women.^275^ Interestingly, endometrial NK cells from HHV-6A-positive infertile women displayed higher cytotoxity toward HHV-6A-infected endometrial epithelial cells and increased levels of surface CXCR3, CX3CR1, CCR2.^276,277^ Patients with spontaneous abortion had four times higher titers of anti-HHV-6 IgG and IgM in comparison with control pregnant women.^278^ A higher percentage of miscarriages and premature deliveries were observed in pregnant patients with pityriasis rosea, which may be related to the reactivation of endogenous HHV-6 or HHV-7. HHV-6/7 DNA was detected in plasma, placenta and/or fetal tissues of 36% of patients with adverse pregnancy outcomes.^279,280^ Shedding of HHV-6 /7 during pregnancy and vertical transmission of HHV-6 have also been reported.^281–284^ However, it is not clear whether HHV-6 actually crosses the placenta, as chromosomal integration in the fetus can also occur. Inherited chromosomally integrated (ici) HHV-6 can indeed reactivate and cause harm to the fetus and newborn.^285,286^ Since HHV-6/7 shedding was associated with a variety of diseases such as multiple sclerosis, chronic fatigue syndrome, AIDS, organ transplant complications, autoimmune diseases, cancer and many others,^287,288^ a substantial challenge is to establish whether HHV-6/7 is actually an etiological factor for these pathologies and not just a harmless satellite finding of virus replication due to loosened immunosurveillance. Nonetheless, recently published data demonstrate that infection of fetuses with iciHHV-6-positive predisposes their mothers to pre-eclampsia independently of the parental origin of iciHHV-6.^289^ Interestingly, HHV-6 infection suppressed angiogenesis and lymphangiogenesis in vitro,^290,291^ and this might affect placenta development. The role of dNK cells in HHV-6 infection during pregnancy is yet to be examined.

## Emerging infections

### SARS-CoV-2

The COVID-19 pandemic had affected more than 38 million people worldwide by the middle of October 2020.^292^ SARS-CoV-2 may affect a variety of physiological and pathological processes, including gestation and pregnancy outcomes. Both SARS-CoV and particularly MERS-CoV, the agents of previous coronavirus pandemics, induce serious pregnancy complications, including intrauterine death, FGR, and maternal death.^293–297^

Although there are some reported cases of SARS-CoV-2 vertical transmission, a thorough investigation is required to confirm this.^298–302^

Other characteristics of COVID-19 pathology may be relevant to possible unfavorable pregnancy courses/outcomes. For example, COVID-19 associates with hypercoagulability and endotheliopathy,^303,304^ which can potentially impact placental blood flow. Although histopathological changes in the placenta of SARS-CoV-2 infected women have been described, their causal association with SARS-CoV-2 infection is unclear.^299,305,306^ The cytokine storm characteristic of some cases of COVID-19,^307,308^ might indirectly mediate pregnancy complications and fetal developmental pathology.^49,50^

A recent meta-analysis and a systematic review suggests good outcomes and no immediate concern for SARS-CoV-2 infections in pregnancy, although a high degree of statistical heterogeneity of the data makes it difficult to draw definitive conclusions^309^ and cases with pregnancy complications are likely underreported. Studies on large cohorts of SARS-CoV-2 positive pregnant women with long-term follow-up of the babies are required.

While dNK cells during SASR-CoV-2 infection have not been investigated yet, numbers of pbNK cells dropped significantly in SARS-CoV-2-infected patients and this correlated with disease severity.^310–314^ Within patients pbNK cells, the proportion of NKG2A^+^ NK cells increased, and expression of intracellular CD107a and IFN-ɣ in total pbNK cells were lower.^314^

## Concluding remarks and future directions

There are still gaps in our knowledge of both systemic and local immune responses to pathogens during pregnancies. For example, while the changes in symptoms of rheumatoid arthritis and multiple sclerosis during pregnancy clearly illustrate the immunological alterations occurring during gestation, how exactly these changes affect the outcome of pregnancy during infections is unknown. However, new technologies such as mass cytometry and single-cell RNA sequencing, allow the generation of algorithms that register the chronology of immunological changes during pregnancy. Deviations from the norm can then be measured during infections or other pregnancy complications to capture the pathogenesis of certain infectious diseases in pregnancy. Physiological changes during gestation may affect the course of an infectious disease even if the immune response is not altered. For example, respiratory viruses may cause more harm in pregnant women due to changes in pulmonary functions in gestation. Placental malaria discussed above is also a case in point. Mass cytometry and single-cell RNA sequencing should help to generate a map of the complexity of dNK-cell heterogeneity at the maternal-fetal interface. The challenge is to determine the role of each cellular subset. In mice, for example, it appears that tissue-resident NK cells may be responsible for fetal growth, while blood-like conventional NK cells may regulate vascular changes by producing IFN-γ.^315^ In humans, dNK1 may engage with trophoblast through KIR. A subset of NKG2C^+^ dNK1 may provide benefits in secondary pregnancy through immunological training,^33^ while dNK2 and dNK3 cells may engage with other cell types in the decidua through the production of other factors such as XCL-1, which can attract both DCs and trophoblast.^316^ As inflammation and infections are associated with abnormal placental vascular development^317,318^ and dNK cells play a significant role in placental vascularization, one of the remaining important research questions is how placental and systemic infection can influence the vascular remodeling function of dNK cells.

The relative roles of NK and CD8^+^ T cells are also difficult to discern, as these cell types share many features. However, temporal changes in the frequencies of the two types of cells in the decidua suggest that tissue-resident T cells may be more important in late gestation, while NK cells may specialize in certain defense mechanisms, like the recently reported nanotube-mediated transfer of granulysin into Listeria-infected trophoblast.^148^ Decidual CD8^+^ T cells are able to degranulate and produce pro-inflammatory cytokines in response to stimulation and are therefore competent in their response to pathogen invasion.^319^ How decidual NK and T cells operate during infections and which cells they interact with can be inferred by looking at the sets of MHC class I molecules expressed on cells in the decidua. Stromal cells express HLA-A, -B -C and -E, and so are capable of both presenting antigens to CD8^+^ T cells and engaging with certain receptors on NK cells, like KIRs, NKG2A, or NKG2C. Trophoblast instead expresses only selected MHC molecules, if any. So, while most trophoblast express no HLA molecules at all, invasive trophoblast cells express HLA-C, a known ligand for KIRs that can also present antigens to CD8^+^ T cells. Invasive trophoblast cells also express HLA-E and HLA-G, which respectively engage NK receptors NKG2A and NKG2C or LILRB1 and KIR2DL4.

Another open question is the presence of a microbiome in the placenta. This is a controversial area and, while the placenta is indeed sterile,^320^ colonization of the fetal intestine by microbial products may be a physiological process that could be affected by pathogens during pregnancy. Finally, another fascinating question is how endogenous retroviruses, with many of their genes expressed in the placenta and some having impacted its evolution,^321^ may engage with immune cells in the decidua and thereby influence immune responses to pathogens during pregnancy.

In this review, we have discussed the ambivalent role of NK-cell immunity to different pathogens in pregnancy outcomes. A comprehensive investigation and understanding of which immune responses are detrimental or, conversely, beneficial for mother and offspring health are critical for further therapeutic development and the improvement of clinical management of pregnant women with infectious diseases.

## References

[1] Van Rood JJ, Eernisse JG, Vam Leeuwen A (1958). Leucocyte antibodies in sera from pregnant women. Nature.

[2] James E (2003). Multiparity induces priming to male-specific minor histocompatibility antigen, HY, in mice and humans. Blood.

[3] Aghaeepour N (2017). An immune clock of human pregnancy. Sci. Immunol..

[4] Kraus TA (2012). Characterizing the pregnancy immune phenotype: results of the viral immunity and pregnancy (VIP) study. J. Clin. Immunol..

[5] Mancini M, Vidal SM (2020). Mechanisms of natural killer cell evasion through viral adaptation. Annu. Rev. Immunol..

[6] Hammer Q, Rückert T, Romagnani C (2018). Natural killer cell specificity for viral infections. Nat. Immunol..

[7] Naiyer MM (2017). KIR2DS2 recognizes conserved peptides derived from viral helicases in the context of HLA-C.. Sci. Immunol..

[8] Sim MJW (2019). Human NK cell receptor KIR2DS4 detects a conserved bacterial epitope presented by HLA-C. Proc. Natl Acad. Sci. USA.

[9] Goodridge JP (2019). Remodeling of secretory lysosomes during education tunes functional potential in NK cells. Nat. Commun..

[10] Bonagura VR (2010). Activating killer cell immunoglobulin-like receptors 3DS1 and 2DS1 protect against developing the severe form of recurrent respiratory papillomatosis. Hum. Immunol..

[11] Cook M (2006). Donor KIR genotype has a major influence on the rate of cytomegalovirus reactivation following T-cell replete stem cell transplantation. Blood.

[12] De Re,V (2015). Genetic diversity of the KIR/HLA system and susceptibility to hepatitis C virus-related diseases. PLoS ONE.

[13] Jennes W (2006). Cutting edge: resistance to HIV-1 infection among African female sex workers is associated with inhibitory KIR in the absence of their HLA ligands. J. Immunol..

[14] Jiang Y (2013). KIR3DS1/L1 and HLA-Bw4-80I are associated with HIV disease progression among HIV typical progressors and long-term nonprogressors. BMC Infect. Dis..

[15] Martin MP (2002). Epistatic interaction between KIR3DS1 and HLA-B delays the progression to AIDS. Nat. Genet..

[16] Khakoo SI (2004). HLA and NK cell inhibitory receptor genes in resolving hepatitis C virus infection. Science.

[17] Moffett A, Colucci F (2014). Uterine NK cells: active regulators at the maternal-fetal interface. J. Clin. Investig..

[18] Moffett A, Colucci F (2015). Co-evolution of NK receptors and HLA ligands in humans is driven by reproduction. Immunol. Rev..

[19] Hiby SE (2010). Maternal activating KIRs protect against human reproductive failure mediated by fetal HLA-C2. J. Clin. Investig..

[20] Xiong S (2013). Maternal uterine NK cell-activating receptor KIR2DS1 enhances placentation. J. Clin. Investig..

[21] Crespo ÂC, van der Zwan A, Ramalho-Santos J, Strominger JL, Tilburgs T (2017). Cytotoxic potential of decidual NK cells and CD8+ T cells awakened by infections. J. Reprod. Immunol..

[22] Crespo ÂC, Strominger JL, Tilburgs T (2016). Expression of KIR2DS1 by decidual natural killer cells increases their ability to control placental HCMV infection. Proc. Natl Acad. Sci. USA.

[23] van der Ploeg K (2017). Modulation of human leukocyte antigen-C by human cytomegalovirus stimulates KIR2DS1 recognition by natural killer cells. Front. Immunol..

[24] Ferreira LMR, Meissner TB, Tilburgs T, Strominger JL (2017). HLA-G: at the interface of Maternal-fetal tolerance. Trends Immunol..

[25] Amiot L, Vu N, Samson M (2014). Immunomodulatory properties of HLA-G in infectious diseases. J. Immunol. Res..

[26] Lin A, Yan WH (2018). Heterogeneity of HLA-G expression in cancers: facing the challenges. Front. Immunol..

[27] Robert-Gangneux F (2011). High level of soluble HLA-G in amniotic fluid is correlated with congenital transmission of *Toxoplasma gondii*. Clin. Immunol..

[28] Münz C (1997). Human histocompatibility leukocyte antigen (HLA)-G molecules inhibit NKAT3 expressing natural killer cells. J. Exp. Med..

[29] Rouas-Freiss N, Marchal RE, Kirszenbaum M, Dausset J, Carosella ED (1997). The alpha1 domain of HLA-G1 and HLA-G2 inhibits cytotoxicity induced by natural killer cells: is HLA-G the public ligand for natural killer cell inhibitory receptors. Proc. Natl Acad. Sci. USA.

[30] Li C, Houser BL, Nicotra ML, Strominger JL (2009). HLA-G homodimer-induced cytokine secretion through HLA-G receptors on human decidual macrophages and natural killer cells. Proc. Natl Acad. Sci. USA.

[31] Rajagopalan S (2006). Activation of NK cells by an endocytosed receptor for soluble HLA-G. PLoS Biol..

[32] Rajagopalan S, Long EO (2012). Cellular senescence induced by CD158d reprograms natural killer cells to promote vascular remodeling. Proc. Natl Acad. Sci. USA.

[33] Gamliel M (2018). Trained memory of human uterine NK cells enhances their function in subsequent pregnancies. Immunity.

[34] King A (2000). HLA-E is expressed on trophoblast and interacts with CD94/NKG2 receptors on decidual NK cells. Eur. J. Immunol..

[35] Shreeve, N. et al. The CD94/NKG2A inhibitory receptor educates uterine NK cells to optimise pregnancy outcomes in humans and mice. *Immunity* - to be published online 21 April 2021. 10.1016/j.immuni.2021.03.021 PMC8211638 33887202

[36] Barrow AD, Martin CJ, Colonna M (2019). The natural cytotoxicity receptors in health and disease. Front. Immunol..

[37] Mandelboim O (2001). Recognition of haemagglutinins on virus-infected cells by NKp46 activates lysis by human NK cells. Nature.

[38] Siewiera J (2015). Natural cytotoxicity receptor splice variants orchestrate the distinct functions of human natural killer cell subtypes. Nat. Commun..

[39] El Costa H (2008). Critical and differential roles of NKp46- and NKp30-activating receptors expressed by uterine NK cells in early pregnancy. J. Immunol..

[40] Gazit R (2006). Lethal influenza infection in the absence of the natural killer cell receptor gene Ncr1. Nat. Immunol..

[41] Felker AM, Chen Z, Foster WG, Croy BA (2013). Receptors for non-MHC ligands contribute to uterine natural killer cell activation during pregnancy in mice. Placenta.

[42] Apps R, Gardner L, Traherne J, Male V, Moffett A (2008). Natural-killer cell ligands at the maternal-fetal interface: UL-16 binding proteins, MHC class-I chain related molecules, HLA-F and CD48. Hum. Reprod..

[43] Hanna J (2006). Decidual NK cells regulate key developmental processes at the human fetal-maternal interface. Nat. Med..

[44] Mincheva-Nilsson L (2006). Placenta-derived soluble MHC class I chain-related molecules down-regulate NKG2D receptor on peripheral blood mononuclear cells during human pregnancy: a possible novel immune escape mechanism for fetal survival. J. Immunol..

[45] Carayannopoulos LN, Barks JL, Yokoyama WM, Riley JK (2010). Murine trophoblast cells induce NK cell interferon-gamma production through KLRK1. Biol. Reprod..

[46] Wight A, Parsons BD, Rahim MMA, Makrigiannis AP (2020). A central role for Ly49 receptors in NK cell memory. J. Immunol..

[47] Gumá M (2004). Imprint of human cytomegalovirus infection on the NK cell receptor repertoire. Blood.

[48] Sun JC, Beilke JN, Lanier LL (2009). Adaptive immune features of natural killer cells. Nature.

[49] Robertson SA, Chin PY, Femia JG, Brown HM (2018). Embryotoxic cytokines-potential roles in embryo loss and fetal programming. J. Reprod. Immunol..

[50] Yockey LJ, Iwasaki A (2018). Interferons and proinflammatory cytokines in pregnancy and fetal development. Immunity.

[51] Li ZY (2014). IFN-γ induces aberrant CD49b^+^ NK cell recruitment through regulating CX3CL1: a novel mechanism by which IFN-γ provokes pregnancy failure. Cell Death Dis..

[52] Thaxton JE (2013). NKG2D blockade inhibits poly(I:C)-triggered fetal loss in wild type but not in IL-10-/- mice. J. Immunol..

[53] Haddad EK, Duclos AJ, Antecka E, Lapp WS, Baines MG (1997). Role of interferon-gamma in the priming of decidual macrophages for nitric oxide production and early pregnancy loss. Cell Immunol..

[54] Murphy SP (2009). Evidence for participation of uterine natural killer cells in the mechanisms responsible for spontaneous preterm labor and delivery. Am. J. Obstet. Gynecol..

[55] Yougbaré I (2017). Activated NK cells cause placental dysfunction and miscarriages in fetal alloimmune thrombocytopenia. Nat. Commun..

[56] Laird, S. M., Lash, G. E., Li, T. C. & Bulmer, J. N. *The Role of Natural Killer Cells in Human Fertility (Scientific Impact Paper No. 53)* (Royal College of Obstetricians and Gynaecologists, 2016).

[57] Dunn D (1999). Mother-to-child transmission of toxoplasmosis: risk estimates for clinical counselling. Lancet.

[58] Foulon W (1999). Treatment of toxoplasmosis during pregnancy: a multicenter study of impact on fetal transmission and children’s sequelae at age 1 year. Am. J. Obstet. Gynecol..

[59] Gómez-Chávez F (2019). Maternal immune response during pregnancy and vertical transmission in human toxoplasmosis. Front. Immunol..

[60] Goldszmid RS (2007). TAP-1 indirectly regulates CD4+ T cell priming in *Toxoplasma gondii* infection by controlling NK cell IFN-gamma production. J. Exp. Med..

[61] Goldszmid RS (2012). NK cell-derived interferon-γ orchestrates cellular dynamics and the differentiation of monocytes into dendritic cells at the site of infection. Immunity.

[62] Guan H, Moretto M, Bzik DJ, Gigley J, Khan IA (2007). NK cells enhance dendritic cell response against parasite antigens via NKG2D pathway. J. Immunol..

[63] Suzuki Y, Orellana MA, Schreiber RD, Remington JS (1988). Interferon-gamma: the major mediator of resistance against *Toxoplasma gondii*. Science.

[64] Denkers EY, Gazzinelli RT, Martin D, Sher A (1993). Emergence of NK1.1+ cells as effectors of IFN-gamma dependent immunity to *Toxoplasma gondii* in MHC class I-deficient mice. J. Exp. Med..

[65] Park E (2019). Toxoplasma gondii infection drives conversion of NK cells into ILC1- like cells. Elife.

[66] Singhania A (2019). Transcriptional profiling unveils type I and II interferon networks in blood and tissues across diseases. Nat. Commun..

[67] Zhang L (2015). Interferon gamma is involved in apoptosis of trophoblast cells at the maternal-fetal interface following Toxoplasma gondii infection. Int. J. Infect. Dis..

[68] Zhao M (2013). IL-10 reduces levels of apoptosis in Toxoplasma gondii-infected trophoblasts. PLoS ONE.

[69] Hunter CA, Subauste CS, Van Cleave VH, Remington JS (1994). Production of gamma interferon by natural killer cells from *Toxoplasma gondii*-infected SCID mice: regulation by interleukin-10, interleukin-12, and tumor necrosis factor alpha. Infect. Immun..

[70] Liu X (2014). Toxoplasma gondii infection of decidual CD1c(+) dendritic cells enhances cytotoxicity of decidual natural killer cells. Inflammation.

[71] Liu CH (2006). Cutting edge: dendritic cells are essential for in vivo IL-12 production and development of resistance against Toxoplasma gondii infection in mice. J. Immunol..

[72] Xu X (2013). Toxoplasma gondii infection regulates the balance of activating and inhibitory receptors on decidual natural killer cells. PLoS ONE.

[73] Hatter JA (2018). Toxoplasma gondii infection triggers chronic cachexia and sustained commensal dysbiosis in mice. PLoS ONE.

[74] Remington, J. S., Klein, J. O. & Wilson, C. *Infectious Diseases of the Fetus and Newborn Infant*. 6th. ed. VI 252–253 (Elsevier, Amsterdam, 2006).

[75] Szabo EK, Finney CAM (2017). *Toxoplasma gondii*: one organism, multiple models. Trends Parasitol..

[76] Abou-Bacar A (2004). Role of gamma interferon and T cells in congenital Toxoplasma transmission. Parasite Immunol..

[77] Scharton-Kersten TM (1996). In the absence of endogenous IFN-gamma, mice develop unimpaired IL-12 responses to Toxoplasma gondii while failing to control acute infection. J. Immunol..

[78] Shiono Y (2007). Maternal-fetal transmission of *Toxoplasma gondii* in interferon-gamma deficient pregnant mice. Parasitol. Int..

[79] Schulthess J (2012). Interleukin-15-dependent NKp46+ innate lymphoid cells control intestinal inflammation by recruiting inflammatory monocytes. Immunity.

[80] Orellana MA, Suzuki Y, Araujo F, Remington JS (1991). Role of beta interferon in resistance to Toxoplasma gondii infection. Infect. Immun..

[81] Denkers EY (1997). Perforin-mediated cytolysis plays a limited role in host resistance to Toxoplasma gondii. J. Immunol..

[82] Hauser WE, Sharma SD, Remington JS (1983). Augmentation of NK cell activity by soluble and particulate fractions of *Toxoplasma gondii*. J. Immunol..

[83] Sharma SD, Verhoef J, Remington JS (1984). Enhancement of human natural killer cell activity by subcellular components of *Toxoplasma gondii*. Cell Immunol..

[84] Senegas A (2009). Toxoplasma gondii-induced foetal resorption in mice involves interferon-gamma-induced apoptosis and spiral artery dilation at the maternofoetal interface. Int J. Parasitol..

[85] Wang C (2018). Toxo*plasma* Chinese 1 Strain of WH3Δrop16_I/III_/gra15_II_ genetic background contributes to abnormal pregnant outcomes in murine model. Front. Immunol..

[86] Xu X (2017). TGF-β1 improving abnormal pregnancy outcomes induced by Toxoplasma gondii infection: Regulating NKG2D/DAP10 and killer subset of decidual NK cells. Cell Immunol..

[87] Zhao M (2017). The effect of TGF-β on Treg cells in adverse pregnancy outcome upon *Toxoplasma gondii* Infection. Front. Microbiol..

[88] Perona-Wright G (2009). Systemic but not local infections elicit immunosuppressive IL-10 production by natural killer cells. Cell Host Microbe.

[89] Wagage S (2014). The aryl hydrocarbon receptor promotes IL-10 production by NK cells. J. Immunol..

[90] Klose CSN (2014). Differentiation of type 1 ILCs from a common progenitor to all helper-like innate lymphoid cell lineages. Cell.

[91] Ivanova DL, Fatima R, Gigley JP (2016). Comparative analysis of conventional natural killer cell responses to acute infection with *Toxoplasma gondii* strains of different virulence. Front. Immunol..

[92] Fell DB (2017). Influenza epidemiology and immunization during pregnancy: final report of a World Health Organization working group. Vaccine.

[93] Jamieson DJ (2009). H1N1 2009 influenza virus infection during pregnancy in the USA. Lancet.

[94] Mertz D (2017). Pregnancy as a risk factor for severe outcomes from influenza virus infection: a systematic review and meta-analysis of observational studies. Vaccine.

[95] Neuzil KM, Reed GW, Mitchel EF, Simonsen L, Griffin MR (1998). Impact of influenza on acute cardiopulmonary hospitalizations in pregnant women. Am. J. Epidemiol..

[96] Beigel JH (2005). Avian influenza A (H5N1) infection in humans. N. Engl. J. Med..

[97] Bradley-Stewart A (2013). Cytokine responses in patients with mild or severe influenza A(H1N1)pdm09. J. Clin. Virol..

[98] Hayden FG (1998). Local and systemic cytokine responses during experimental human influenza A virus infection. Relation to symptom formation and host defense. J. Clin. Investig..

[99] Liu Q, Zhou YH, Yang ZQ (2016). The cytokine storm of severe influenza and development of immunomodulatory therapy. Cell Mol. Immunol..

[100] Denney L (2010). Reduction of natural killer but not effector CD8 T lymphocytes in three consecutive cases of severe/lethal H1N1/09 influenza A virus infection. PLoS ONE.

[101] Lin SJ (2012). Effect of influenza A infection on umbilical cord blood natural killer function regulation with interleukin-15. J. Infect. Dis..

[102] Mao H (2010). Inhibition of human natural killer cell activity by influenza virions and hemagglutinin. J. Virol..

[103] Le Gars M (2019). Pregnancy-induced alterations in NK cell phenotype and function. Front. Immunol..

[104] Kay AW (2014). Enhanced natural killer-cell and T-cell responses to influenza A virus during pregnancy. Proc. Natl Acad. Sci. USA.

[105] Chan KH (2010). Wild type and mutant 2009 pandemic influenza A (H1N1) viruses cause more severe disease and higher mortality in pregnant BALB/c mice. PLoS ONE.

[106] Marcelin G (2011). Fatal outcome of pandemic H1N1 2009 influenza virus infection is associated with immunopathology and impaired lung repair, not enhanced viral burden, in pregnant mice. J. Virol..

[107] Williams K, Mackenzie JS (1977). Influenza infections during pregnancy in the mouse. J. Hyg..

[108] Pazos MA, Kraus TA, Muñoz-Fontela C, Moran TM (2012). Estrogen mediates innate and adaptive immune alterations to influenza infection in pregnant mice. PLoS ONE.

[109] Cappelletti M (2017). Type I interferons regulate susceptibility to inflammation-induced preterm birth. JCI Insight.

[110] Cappelletti M, Della Bella S, Ferrazzi E, Mavilio D, Divanovic S (2016). Inflammation and preterm birth. J. Leukoc. Biol..

[111] Jamieson AM (2013). Role of tissue protection in lethal respiratory viral-bacterial coinfection. Science.

[112] Racicot K (2013). Viral infection of the pregnant cervix predisposes to ascending bacterial infection. J. Immunol..

[113] Abdul-Careem MF (2012). Critical role of natural killer cells in lung immunopathology during influenza infection in mice. J. Infect. Dis..

[114] Nakamura R (2010). Interleukin-15 is critical in the pathogenesis of influenza a virus-induced acute lung injury. J. Virol..

[115] Zhou G, Juang SW, Kane KP (2013). NK cells exacerbate the pathology of influenza virus infection in mice. Eur. J. Immunol..

[116] Kourtis AP, Read JS, Jamieson DJ (2014). Pregnancy and infection. N. Engl. J. Med..

[117] Shulman CE, Dorman EK (2003). Importance and prevention of malaria in pregnancy. Trans. R. Soc. Trop. Med. Hyg..

[118] Umbers AJ, Aitken EH, Rogerson SJ (2011). Malaria in pregnancy: small babies, big problem. Trends Parasitol..

[119] Rogerson SJ, Hviid L, Duffy PE, Leke RF, Taylor DW (2007). Malaria in pregnancy: pathogenesis and immunity. Lancet Infect. Dis..

[120] Stevenson MM, Riley EM (2004). Innate immunity to malaria. Nat. Rev. Immunol..

[121] Tukwasibwe S (2020). Variations in killer-cell immunoglobulin-like receptor and human leukocyte antigen genes and immunity to malaria. Cell. Mol. Immunol..

[122] Wolf AS, Sherratt S, Riley EM (2017). NK cells: uncertain allies against malaria. Front. Immunol..

[123] Arora G (2018). NK cells inhibit Plasmodium falciparum growth in red blood cells via antibody-dependent cellular cytotoxicity. Elife.

[124] Korbel DS, Newman KC, Almeida CR, Davis DM, Riley EM (2005). Heterogeneous human NK cell responses to *Plasmodium falciparum*-infected erythrocytes. J. Immunol..

[125] Artavanis-Tsakonas K, Riley EM (2002). Innate immune response to malaria: rapid induction of IFN-gamma from human NK cells by live *Plasmodium falciparum*-infected erythrocytes. J. Immunol..

[126] Horowitz A (2010). Cross-talk between T cells and NK cells generates rapid effector responses to *Plasmodium falciparum*-infected erythrocytes. J. Immunol..

[127] Omosun YO (2012). Differential association of gene content polymorphisms of killer cell immunoglobulin-like receptors with placental malaria in HIV- and HIV+ mothers. PLoS ONE.

[128] Artavanis-Tsakonas K, Tongren JE, Riley EM (2003). The war between the malaria parasite and the immune system: immunity, immunoregulation and immunopathology. Clin. Exp. Immunol..

[129] Moormann AM (1999). Malaria and pregnancy: placental cytokine expression and its relationship to intrauterine growth retardation. J. Infect. Dis..

[130] Othoro C (2008). Elevated gamma interferon-producing NK cells, CD45RO memory-like T cells, and CD4 T cells are associated with protection against malaria infection in pregnancy. Infect. Immun..

[131] Davison BB (2006). The role of soluble tumor necrosis factor receptor types I and II and tumor necrosis factor-alpha in malaria during pregnancy. J. Infect. Dis..

[132] Barateiro A, Pereira MLM, Epiphanio S, Marinho CRF (2019). Contribution of murine models to the study of malaria during pregnancy. Front. Microbiol..

[133] Neres R, Marinho CR, Gonçalves LA, Catarino MB, Penha-Gonçalves C (2008). Pregnancy outcome and placenta pathology in Plasmodium berghei ANKA infected mice reproduce the pathogenesis of severe malaria in pregnant women. PLoS ONE.

[134] Favre N, Ryffel B, Bordmann G, Rudin W (1997). The course of *Plasmodium chabaudi* chabaudi infections in interferon-gamma receptor deficient mice. Parasite Immunol..

[135] Su Z, Stevenson MM (2000). Central role of endogenous gamma interferon in protective immunity against blood-stage *Plasmodium chabaudi* AS infection. Infect. Immun..

[136] Poovassery JS, Sarr D, Smith G, Nagy T, Moore JM (2009). Malaria-induced murine pregnancy failure: distinct roles for IFN-gamma and TNF. J. Immunol..

[137] Charlier C, Disson O, Lecuit M (2020). Maternal-neonatal listeriosis. Virulence.

[138] Disson O (2008). Conjugated action of two species-specific invasion proteins for fetoplacental listeriosis. Nature.

[139] Pamer EG (2004). Immune responses to *Listeria monocytogene*s. Nat. Rev. Immunol..

[140] Williams MA, Schmidt RL, Lenz LL (2012). Early events regulating immunity and pathogenesis during *Listeria monocytogenes* infection. Trends Immunol..

[141] Zenewicz LA, Shen H (2007). Innate and adaptive immune responses to Listeria monocytogenes: a short overview. Microbes Infect..

[142] Huang S (1993). Immune response in mice that lack the interferon-gamma receptor. Science.

[143] Reynders A (2011). Identity, regulation and in vivo function of gut NKp46+RORγt+ and NKp46+RORγt- lymphoid cells. EMBO J..

[144] Clark SE (2016). Bacterial Manipulation of NK Cell Regulatory Activity Increases Susceptibility to *Listeria monocytogenes* Infection. PLoS Pathog..

[145] Thäle C, Kiderlen AF (2005). Sources of interferon-gamma (IFN-gamma) in early immune response to *Listeria monocytogenes*. Immunobiology.

[146] Barber EM, Pollard JW (2003). The uterine NK cell population requires IL-15 but these cells are not required for pregnancy nor the resolution of a *Listeria monocytogenes* infection. J. Immunol..

[147] Teixeira HC, Kaufmann SH (1994). Role of NK1.1+ cells in experimental listeriosis. NK1+ cells are early IFN-gamma producers but impair resistance to *Listeria monocytogenes* infection. J. Immunol..

[148] Crespo ÂC (2020). Decidual NK cells transfer granulysin to selectively kill bacteria in trophoblasts. Cell.

[149] Maroof A (2008). Posttranscriptional regulation of II10 gene expression allows natural killer cells to express immunoregulatory function. Immunity.

[150] Manns MP (2017). Hepatitis C virus infection. Nat. Rev. Dis. Prim..

[151] Spearman CW, Dusheiko GM, Hellard M, Sonderup M (2019). Hepatitis C. Lancet.

[152] Rehermann B (2009). Hepatitis C virus versus innate and adaptive immune responses: a tale of coevolution and coexistence. J. Clin. Investig..

[153] Rehermann B (2013). Pathogenesis of chronic viral hepatitis: differential roles of T cells and NK cells. Nat. Med..

[154] World Health Organization. *Global Hepatitis Report 2017* (World Health Organization, 2017).

[155] Benova L, Mohamoud YA, Calvert C, Abu-Raddad LJ (2014). Vertical transmission of hepatitis C virus: systematic review and meta-analysis. Clin. Infect. Dis..

[156] Azzari C (2008). Higher risk of hepatitis C virus perinatal transmission from drug user mothers is mediated by peripheral blood mononuclear cell infection. J. Med. Virol..

[157] Prasad MR, Honegger JR (2013). Hepatitis C virus in pregnancy. Am. J. Perinatol..

[158] Steininger C (2003). Increased risk of mother-to-infant transmission of hepatitis C virus by intrapartum infantile exposure to maternal blood. J. Infect. Dis..

[159] Thomas SL, Newell ML, Peckham CS, Ades AE, Hall AJ (1998). A review of hepatitis C virus (HCV) vertical transmission: risks of transmission to infants born to mothers with and without HCV viraemia or human immunodeficiency virus infection. Int. J. Epidemiol..

[160] Zanetti AR, Tanzi E, Newell ML (1999). Mother-to-infant transmission of hepatitis C virus. J. Hepatol..

[161] Gibb DM (2000). Mother-to-child transmission of hepatitis C virus: evidence for preventable peripartum transmission. Lancet.

[162] Mok J, Pembrey L, Tovo PA, Newell ML, European, P. H. C. V. N. (2005). When does mother to child transmission of hepatitis C virus occur. Arch. Dis. Child Fetal Neonatal Ed..

[163] Connell LE (2011). Maternal hepatitis B and hepatitis C carrier status and perinatal outcomes. Liver Int.

[164] Floreani A (2013). Hepatitis C and pregnancy. World J. Gastroenterol..

[165] Huang QT (2016). Maternal HCV infection is associated with intrauterine fetal growth disturbance: A meta-analysis of observational studies. Medicine.

[166] Jabeen T (2000). Pregnancy and pregnancy outcome in hepatitis C type 1b. QJM.

[167] Kumar A, Sharma KA, Gupta RK, Kar P, Chakravarti A (2007). Pregnancy outcome in hepatitis C virus infection. Int J. Gynaecol. Obstet..

[168] Reddick KL, Jhaveri R, Gandhi M, James AH, Swamy GK (2011). Pregnancy outcomes associated with viral hepatitis. J. Viral Hepat..

[169] Salemi JL (2014). Maternal hepatitis B and hepatitis C infection and neonatal neurological outcomes. J. Viral Hepat..

[170] Cacoub P (2016). Extrahepatic manifestations of chronic hepatitis C virus infection. Ther. Adv. Infect. Dis..

[171] Flores-Chávez A, Carrion JA, Forns X, Ramos-Casals M (2017). Extrahepatic manifestations associated with chronic hepatitis C virus infection. Rev. Esp. Sanid. Penit..

[172] Himoto T, Masaki T (2012). Extrahepatic manifestations and autoantibodies in patients with hepatitis C virus infection. Clin. Dev. Immunol..

[173] Palazzi C, D’Amico E, D’Angelo S, Gilio M, Olivieri I (2016). Rheumatic manifestations of hepatitis C virus chronic infection: Indications for a correct diagnosis. World J. Gastroenterol..

[174] Zampino R (2013). Chronic HCV infection and inflammation: Clinical impact on hepatic and extra-hepatic manifestations. World J. Hepatol..

[175] Fasbender F, Widera A, Hengstler JG, Watzl C (2016). Natural killer cells and liver fibrosis. Front. Immunol..

[176] Strunz B (2018). Chronic hepatitis C virus infection irreversibly impacts human natural killer cell repertoire diversity. Nat. Commun..

[177] Yoon JC, Yang CM, Song Y, Lee JM (2016). Natural killer cells in hepatitis C: current progress. World J. Gastroenterol..

[178] Vercauteren K, de Jong YP, Meuleman P (2015). Animal models for the study of HCV. Curr. Opin. Virol..

[179] Fauteux-Daniel S (2017). Vertical transmission of hepatitis C virus: variable transmission bottleneck and evidence of midgestation in utero infection. J. Virol..

[180] Giugliano S (2015). Hepatitis C virus sensing by human trophoblasts induces innate immune responses and recruitment of maternal NK cells: potential implications for limiting vertical transmission. J. Immunol..

[181] Nie QH (2012). Hepatitis C virus infection of human cytotrophoblasts cultured in vitro. J. Med. Virol..

[182] UNAIDS Data 2020. *Joint United Nations Programme on HIV/AIDS* (UNAIDS, 2020). 12349391

[183] Sharp PM, Hahn BH (2011). Origins of HIV and the AIDS pandemic. Cold Spring Harb. Perspect. Med..

[184] Wilen CB, Tilton JC, Doms RW (2012). HIV: cell binding and entry. Cold Spring Harb. Perspect. Med..

[185] De Cock KM (2000). Prevention of mother-to-child HIV transmission in resource-poor countries: translating research into policy and practice. JAMA.

[186] Newell ML (2001). Prevention of mother-to-child transmission of HIV: challenges for the current decade. Bull. World Health Organ..

[187] Cooper ER (2002). Combination antiretroviral strategies for the treatment of pregnant HIV-1-infected women and prevention of perinatal HIV-1 transmission. J. Acquir Immune Defic. Syndr..

[188] Garcia-Tejedor A, Maiques V, Perales A, Lopez-Aldeguer J (2009). Influence of highly active antiretroviral treatment (HAART) on risk factors for vertical HIV transmission. Acta Obstet. Gynecol. Scand..

[189] Siegfried, N., van der Merwe, L., Brocklehurst, P. & Sint, T. T. Antiretrovirals for reducing the risk of mother-to-child transmission of HIV infection. *Cochrane Database Syst. Rev.* CD003510 https://pubmed.ncbi.nlm.nih.gov/21735394/ (2011). 10.1002/14651858.CD003510.pub3 PMC13168846 21735394

[190] Hénin Y (1993). Virus excretion in the cervicovaginal secretions of pregnant and nonpregnant HIV-infected women. J. Acquir Immune Defic. Syndr. (1988).

[191] Lewis P (1998). Cell-free human immunodeficiency virus type 1 in breast milk. J. Infect. Dis..

[192] Kuhn L (1997). Timing of maternal-infant HIV transmission: associations between intrapartum factors and early polymerase chain reaction results. New York City Perinatal HIV Transmission Collaborative Study Group. AIDS.

[193] Minkoff H (1995). The relationship of the duration of ruptured membranes to vertical transmission of human immunodeficiency virus. Am. J. Obstet. Gynecol..

[194] Mock PA (1999). Maternal viral load and timing of mother-to-child HIV transmission, Bangkok, Thailand. Bangkok Collaborative Perinatal HIV Transmission Study Group. AIDS.

[195] Shaffer N (1999). Maternal virus load and perinatal human immunodeficiency virus type 1 subtype E transmission, Thailand. Bangkok Collaborative Perinatal HIV Transmission Study Group. J. Infect. Dis..

[196] Milligan C, Overbaugh J (2014). The role of cell-associated virus in mother-to-child HIV transmission. J. Infect. Dis..

[197] Chandwani S (1991). Pathology and human immunodeficiency virus expression in placentas of seropositive women. J. Infect. Dis..

[198] Temmerman M (1994). Maternal human immunodeficiency virus-1 infection and pregnancy outcome. Obstet. Gynecol..

[199] Schwartz DA (2000). Placental abnormalities associated with human immunodeficiency virus type 1 infection and perinatal transmission in Bangkok, Thailand. J. Infect. Dis..

[200] Maternal viral load and vertical transmission of HIV-1: an important factor but not the only one. (1999). The European Collaborative Study. AIDS.

[201] European MODC (1999). Elective caesarean-section versus vaginal delivery in prevention of vertical HIV-1 transmission: a randomised clinical trial. Lancet.

[202] International PHIVG (1999). The mode of delivery and the risk of vertical transmission of human immunodeficiency virus type 1–a meta-analysis of 15 prospective cohort studies. N. Engl. J. Med..

[203] Abrams EJ (1995). Neonatal predictors of infection status and early death among 332 infants at risk of HIV-1 infection monitored prospectively from birth. New York City Perinatal HIV Transmission Collaborative Study Group. Pediatrics.

[204] Dara JS, Hanna DB, Anastos K, Wright R, Herold BC (2018). Low birth weight in human immunodeficiency virus-exposed uninfected infants in Bronx, New York. J. Pediatr. Infect. Dis. Soc..

[205] Eckard AR, Kirk SE, Hagood NL (2019). Contemporary Issues in Pregnancy (and Offspring) in the current HIV era. Curr. HIV/AIDS Rep..

[206] Ramokolo V (2017). In utero ART exposure and birth and early growth outcomes among HIV-exposed uninfected infants attending immunization services: results from national PMTCT surveillance, South Africa. Open Forum Infect. Dis..

[207] Stringer EM (2018). Pregnancy outcomes among HIV-infected women who conceived on antiretroviral therapy. PLoS ONE.

[208] Uthman OA (2017). Timing of initiation of antiretroviral therapy and adverse pregnancy outcomes: a systematic review and meta-analysis. Lancet HIV.

[209] Flórez-Álvarez L, Hernandez JC, Zapata W (2018). NK cells in HIV-1 infection: from basic science to vaccine strategies. Front. Immunol..

[210] Mikulak J, Oriolo F, Zaghi E, Di Vito C, Mavilio D (2017). Natural killer cells in HIV-1 infection and therapy. AIDS.

[211] Scully E, Alter G (2016). NK Cells in HIV disease. Curr. HIV/AIDS Rep..

[212] Mselle TF, Howell AL, Ghosh M, Wira CR, Sentman CL (2009). Human uterine natural killer cells but not blood natural killer cells inhibit human immunodeficiency virus type 1 infection by secretion of CXCL12. J. Virol..

[213] Quillay H (2016). NK cells control HIV-1 infection of macrophages through soluble factors and cellular contacts in the human decidua. Retrovirology.

[214] Teixeira FME, Pietrobon AJ, Oliveira LM, Oliveira LMDS, Sato MN (2020). Maternal-fetal interplay in Zika virus infection and adverse perinatal outcomes. Front. Immunol..

[215] Shapiro-Mendoza CK (2017). Pregnancy outcomes after maternal Zika virus infection during pregnancy—US Territories, January 1, 2016–April 25, 2017. Morbidity Mortal. Wkly. Rep..

[216] Yockey LJ (2016). Vaginal exposure to Zika virus during pregnancy leads to fetal brain infection. Cell.

[217] El Costa H (2016). ZIKA virus reveals broad tissue and cell tropism during the first trimester of pregnancy. Sci. Rep..

[218] Jurado KA (2016). Zika virus productively infects primary human placenta-specific macrophages. JCI Insight.

[219] Quicke KM (2016). Zika virus infects human placental macrophages. Cell Host Microbe.

[220] Tabata T (2016). Zika virus targets different primary human placental cells, suggesting two routes for vertical transmission. Cell Host Microbe.

[221] Tabata T (2018). Zika virus replicates in proliferating cells in explants from first-trimester human placentas, potential sites for dissemination of infection. J. Infect. Dis..

[222] Sheridan MA (2017). Vulnerability of primitive human placental trophoblast to Zika virus. Proc. Natl Acad. Sci. USA.

[223] Bayer A (2016). Type III interferons produced by human placental trophoblasts confer protection against Zika virus infection. Cell Host Microbe.

[224] Bhatnagar J (2017). Zika virus RNA replication and persistence in brain and placental tissue. Emerg. Infect. Dis..

[225] de Noronha L (2018). Zika virus infection at different pregnancy stages: anatomopathological findings, target cells and viral persistence in placental tissues. Front. Microbiol..

[226] Noronha L, Zanluca C, Azevedo ML, Luz KG, Santos CN (2016). Zika virus damages the human placental barrier and presents marked fetal neurotropism. Mem. Inst. Oswaldo Cruz.

[227] Barros JBS (2018). Acute zika virus infection in an endemic area shows modest proinflammatory systemic immunoactivation and cytokine-symptom associations. Front. Immunol..

[228] Lum FM (2018). Longitudinal study of cellular and systemic cytokine signatures to define the dynamics of a balanced immune environment during disease manifestation in Zika virus-infected patients. J. Infect. Dis..

[229] Tappe D (2016). Cytokine kinetics of Zika virus-infected patients from acute to reconvalescent phase. Med. Microbiol. Immunol..

[230] Maucourant C (2019). Zika virus in the eye of the cytokine storm. Eur. Cytokine Netw..

[231] Ornelas AM (2017). Immune activation in amniotic fluid from Zika virus-associated microcephaly. Ann. Neurol..

[232] Weisblum Y (2017). Zika virus infects early- and midgestation human maternal decidual tissues, inducing distinct innate tissue responses in the maternal-fetal interface. J. Virol..

[233] Glasner A (2017). Zika virus escapes NK cell detection by upregulating major histocompatibility complex class I molecules. J. Virol..

[234] Dudley DM (2016). A rhesus macaque model of Asian-lineage Zika virus infection. Nat. Commun..

[235] Cugola FR (2016). The Brazilian Zika virus strain causes birth defects in experimental models. Nature.

[236] Miner JJ (2016). Zika virus infection during pregnancy in mice causes placental damage and fetal demise. Cell.

[237] Mysorekar IU, Diamond MS (2016). Modeling Zika virus infection in pregnancy. N. Engl. J. Med..

[238] Vermillion MS (2017). Intrauterine Zika virus infection of pregnant immunocompetent mice models transplacental transmission and adverse perinatal outcomes. Nat. Commun..

[239] Banatvala, J. & Peckham, C. *Rubella Viruses* (Elsevier, 2007).

[240] Lambert N, Strebel P, Orenstein W, Icenogle J, Poland GA (2015). Rubella. Lancet.

[241] Berger, S. *Rubella: Global Status: 2019 edition* (GIDEON Informatics Inc, 2019).

[242] Duszak RS (2009). Congenital rubella syndrome–major review. Optometry.

[243] Castejon O (2019). Rubella virus, infecting the placental villi. Int. Med..

[244] Garcia AG, Marques RL, Lobato YY, Fonseca ME, Wigg MD (1985). Placental pathology in congenital rubella. Placenta.

[245] Adamo MP, Zapata M, Frey TK (2008). Analysis of gene expression in fetal and adult cells infected with rubella virus. Virology.

[246] Biron CA, Byron KS, Sullivan JL (1989). Severe herpesvirus infections in an adolescent without natural killer cells. N. Engl. J. Med..

[247] Mace EM, Orange JS (2019). Emerging insights into human health and NK cell biology from the study of NK cell deficiencies. Immunol. Rev..

[248] Fowler KB (1992). The outcome of congenital cytomegalovirus infection in relation to maternal antibody status. N. Engl. J. Med..

[249] Goderis J (2014). Hearing loss and congenital CMV infection: a systematic review. Pediatrics.

[250] Kenneson A, Cannon MJ (2007). Review and meta-analysis of the epidemiology of congenital cytomegalovirus (CMV) infection. Rev. Med. Virol..

[251] Ornoy A, Weinstein-Fudim L, Ergaz Z (2016). Genetic syndromes, maternal diseases and antenatal factors associated with autism spectrum disorders (ASD). Front. Neurosci..

[252] Hughes BL, Gyamfi-Bannerman C, Society for Maternal-Fetal Medicine, S. M. F. M. (2016). Diagnosis and antenatal management of congenital cytomegalovirus infection. Am. J. Obstet. Gynecol..

[253] Pass RF, Fowler KB, Boppana SB, Britt WJ, Stagno S (2006). Congenital cytomegalovirus infection following first trimester maternal infection: symptoms at birth and outcome. J. Clin. Virol..

[254] Jarvis MA, Nelson JA (2007). Human cytomegalovirus tropism for endothelial cells: not all endothelial cells are created equal. J. Virol..

[255] Fisher S, Genbacev O, Maidji E, Pereira L (2000). Human cytomegalovirus infection of placental cytotrophoblasts in vitro and in utero: implications for transmission and pathogenesis. J. Virol..

[256] Gabrielli L (2012). Congenital cytomegalovirus infection: patterns of fetal brain damage. Clin. Microbiol. Infect..

[257] Maidji E, Genbacev O, Chang HT, Pereira L (2007). Developmental regulation of human cytomegalovirus receptors in cytotrophoblasts correlates with distinct replication sites in the placenta. J. Virol..

[258] Weisblum Y (2011). Modeling of human cytomegalovirus maternal-fetal transmission in a novel decidual organ culture. J. Virol..

[259] Pereira L (2014). Intrauterine growth restriction caused by underlying congenital cytomegalovirus infection. J. Infect. Dis..

[260] Siewiera J (2013). Human cytomegalovirus infection elicits new decidual natural killer cell effector functions. PLoS Pathog..

[261] de Mendonça Vieira R (2020). Human term pregnancy decidual NK cells generate distinct cytotoxic responses. J. Immunol..

[262] Tilburgs T, Evans JH, Crespo ÂC, Strominger JL (2015). The HLA-G cycle provides for both NK tolerance and immunity at the maternal-fetal interface. Proc. Natl Acad. Sci. USA.

[263] Riou, R. et al. Severe symptomatic primary human cytomegalovirus infection despite effective innate and adaptive immune responses. *J. Virol*. **91** (2017). 10.1128/JVI.02245-16 PMC5309965 28031361

[264] Scott GM (2012). Cytomegalovirus infection during pregnancy with maternofetal transmission induces a proinflammatory cytokine bias in placenta and amniotic fluid. J. Infect. Dis..

[265] Weisblum Y (2015). Human cytomegalovirus induces a distinct innate immune response in the maternal-fetal interface. Virology.

[266] Chan G, Hemmings DG, Yurochko AD, Guilbert LJ (2002). Human cytomegalovirus-caused damage to placental trophoblasts mediated by immediate-early gene-induced tumor necrosis factor-alpha. Am. J. Pathol..

[267] Fox, J. G. *The Mouse in Biomedical Research* (Elsevier, 2007).

[268] Gombos RB, Wolan V, McDonald K, Hemmings DG (2009). Impaired vascular function in mice with an active cytomegalovirus infection. Am. J. Physiol. Heart Circ. Physiol..

[269] Liao Y (2018). Maternal murine cytomegalovirus infection during pregnancy up-regulates the gene expression of toll-like receptor 2 and 4 in placenta. Curr. Med. Sci..

[270] De Pelsmaeker S, Romero N, Vitale M, Favoreel HW (2018). Herpesvirus evasion of natural killer cells. J. Virol..

[271] Della Chiesa M (2019). Human NK cells and herpesviruses: mechanisms of recognition, response and adaptation. Front. Microbiol..

[272] Kapranos NC, Kotronias DC (2009). Detection of herpes simplex virus in first trimester pregnancy loss using molecular techniques. In Vivo.

[273] Kim ID, Chang HS, Hwang KJ (2012). Herpes simplex virus 2 infection rate and necessity of screening during pregnancy: a clinical and seroepidemiologic study. Yonsei Med. J..

[274] Ashkar AA, Rosenthal KL (2003). Interleukin-15 and natural killer and NKT cells play a critical role in innate protection against genital herpes simplex virus type 2 infection. J. Virol..

[275] Marci R (2016). Presence of HHV-6A in endometrial epithelial cells from women with primary unexplained infertility. PLoS ONE.

[276] Caselli E (2017). HHV-6A infection of endometrial epithelial cells induces increased endometrial NK cell-mediated cytotoxicity. Front. Microbiol..

[277] Eliassen E, Di Luca D, Rizzo R, Barao I (2017). The interplay between natural killer cells and human herpesvirus-6. Viruses.

[278] Ando Y, Kakimoto K, Ekuni Y, Ichijo M (1992). HHV-6 infection during pregnancy and spontaneous abortion. Lancet.

[279] Drago F (2008). Pregnancy outcome in patients with pityriasis rosea. J. Am. Acad. Dermatol..

[280] Drago F (2014). Evidence of human herpesvirus-6 and -7 reactivation in miscarrying women with pityriasis rosea. J. Am. Acad. Dermatol..

[281] Caserta MT, Hall CB, Schnabel K, Lofthus G, McDermott MP (2007). Human herpesvirus (HHV)-6 and HHV-7 infections in pregnant women. J. Infect. Dis..

[282] Caserta MT (2014). Early developmental outcomes of children with congenital HHV-6 infection. Pediatrics.

[283] Dahl H (1999). Reactivation of human herpesvirus 6 during pregnancy. J. Infect. Dis..

[284] Ohashi M (2002). Reactivation of human herpesvirus 6 and 7 in pregnant women. J. Med Virol..

[285] Arbuckle JH (2010). The latent human herpesvirus-6A genome specifically integrates in telomeres of human chromosomes in vivo and in vitro. Proc. Natl Acad. Sci. USA.

[286] Das BB (2016). Possible progesterone-induced gestational activation of chromosomally integrated human herpesvirus 6B and transplacental transmission of activated human herpesvirus 6B. J. Heart Lung Transpl..

[287] Agut H, Bonnafous P, Gautheret-Dejean A (2017). Update on infections with human herpesviruses 6A, 6B, and 7. Med Mal. Infect..

[288] Clark DA (2000). Human herpesvirus 6. Rev. Med. Virol..

[289] Gaccioli F (2020). Fetal inheritance of chromosomally integrated human herpesvirus 6 predisposes the mother to pre-eclampsia. Nat. Microbiol..

[290] Caruso A (2009). U94 of human herpesvirus 6 inhibits in vitro angiogenesis and lymphangiogenesis. Proc. Natl Acad. Sci. USA.

[291] Rizzo R (2018). Human Herpesvirus 6A and 6B inhibit in vitro angiogenesis by induction of Human Leukocyte Antigen G. Sci. Rep..

[292] WHO. *Coronavirus Disease (COVID-19) Situation Reports* (WHO, 2020).

[293] Assiri A (2016). Middle east respiratory syndrome coronavirus infection during pregnancy: a report of 5 cases from Saudi Arabia. Clin. Infect. Dis..

[294] de Souza Silva GA (2020). SARS-CoV, MERS-CoV and SARS-CoV-2 infections in pregnancy and fetal development. J. Gynecol. Obstet. Hum. Reprod..

[295] Malik A, El Masry KM, Ravi M, Sayed F (2016). Middle east respiratory syndrome coronavirus during pregnancy, Abu Dhabi, United Arab Emirates, 2013. Emerg. Infect. Dis..

[296] Ng WF (2006). The placentas of patients with severe acute respiratory syndrome: a pathophysiological evaluation. Pathology.

[297] Payne DC (2014). Stillbirth during infection with Middle East respiratory syndrome coronavirus. J. Infect. Dis..

[298] Galang RR (2020). Severe coronavirus infections in pregnancy: a systematic review. Obstet. Gynecol..

[299] Mahyuddin, A. P. et al. Mechanisms and evidence of vertical transmission of infections in pregnancy including SARS-CoV-2. *Prenat. Diagn.*10.1002/pd.5765 (2020). [Epub ahead of print] 10.1002/pd.5765 PMC7307070 32529643

[300] Simões E Silva AC, Leal CRV (2020). Is SARS-CoV-2 vertically transmitted. Front. Pediatr..

[301] Algarroba GN (2020). Visualization of severe acute respiratory syndrome coronavirus 2 invading the human placenta using electron microscopy. Am. J. Obstet. Gynecol..

[302] Hosier H (2020). SARS-CoV-2 infection of the placenta. J. Clin. Investig..

[303] Connors JM, Levy JH (2020). COVID-19 and its implications for thrombosis and anticoagulation. Blood.

[304] Varga Z (2020). Endothelial cell infection and endotheliitis in COVID-19. Lancet.

[305] Baergen RN, Heller DS (2020). Placental pathology in Covid-19 positive mothers: preliminary findings. Pediatr. Dev. Pathol..

[306] Shanes ED (2020). Placental pathology in COVID-19. Am. J. Clin. Pathol..

[307] Blanco-Melo D (2020). Imbalanced host response to SARS-CoV-2 drives development of COVID-19. Cell.

[308] Chen G (2020). Clinical and immunological features of severe and moderate coronavirus disease 2019. J. Clin. Investig..

[309] Khalil, A. et al. SARS-CoV-2 infection in pregnancy: a systematic review and meta-analysis of clinical features and pregnancy outcomes. *EClinicalMedicine* 100446 (2020). 10.1016/j.eclinm.2020.100446 PMC7334039 32838230

[310] Giamarellos-Bourboulis EJ (2020). Complex immune dysregulation in COVID-19 patients with severe respiratory failure. Cell Host Microbe.

[311] Qin C (2020). Dysregulation of immune response in patients with coronavirus 2019 (COVID-19) in Wuhan, China. Clin. Infect. Dis..

[312] Wen W (2020). Immune cell profiling of COVID-19 patients in the recovery stage by single-cell sequencing. Cell Discov..

[313] Wilk AJ (2020). A single-cell atlas of the peripheral immune response in patients with severe COVID-19. Nat. Med..

[314] Zheng M (2020). Functional exhaustion of antiviral lymphocytes in COVID-19 patients. Cell Mol. Immunol..

[315] Filipovic I (2018). Molecular definition of group 1 innate lymphoid cells in the mouse uterus. Nat. Commun..

[316] Huhn O (2020). Distinctive phenotypes and functions of innate lymphoid cells in human decidua during early pregnancy. Nat. Commun..

[317] Conroy AL (2013). Complement activation and the resulting placental vascular insufficiency drives fetal growth restriction associated with placental malaria. Cell Host Microbe.

[318] Weckman AM, Ngai M, Wright J, McDonald CR, Kain KC (2019). The impact of infection in pregnancy on placental vascular development and adverse birth outcomes. Front. Microbiol..

[319] van der Zwan A (2018). Mixed signature of activation and dysfunction allows human decidual CD8^+^ T cells to provide both tolerance and immunity. Proc. Natl Acad. Sci. USA.

[320] de Goffau MC (2019). Human placenta has no microbiome but can contain potential pathogens. Nature.

[321] Mi S (2000). Syncytin is a captive retroviral envelope protein involved in human placental morphogenesis. Nature.

